# Colorectal cancer risk following polypectomy in a multicentre, retrospective, cohort study: an evaluation of the 2020 UK post-polypectomy surveillance guidelines

**DOI:** 10.1136/gutjnl-2020-323411

**Published:** 2021-03-05

**Authors:** Amanda J Cross, Emma C Robbins, Kevin Pack, Iain Stenson, Bhavita Patel, Matthew D Rutter, Andrew M Veitch, Brian P Saunders, Stephen W Duffy, Kate Wooldrage

**Affiliations:** 1 Cancer Screening and Prevention Research Group (CSPRG), Department of Surgery and Cancer, Imperial College London, London, UK; 2 Department of Gastroenterology, University Hospital of North Tees, Stockton-on-Tees, UK; 3 Faculty of Medical Sciences, Newcastle University, Newcastle-upon-Tyne, UK; 4 Department of Gastroenterology, New Cross Hospital, Wolverhampton, UK; 5 Wolfson Unit for Endoscopy, St Mark's Hospital, Harrow, London, UK; 6 Centre for Cancer Prevention, Wolfson Institute of Preventive Medicine, Queen Mary University of London, London, UK

**Keywords:** colorectal adenomas, colorectal cancer, colonoscopy, colorectal cancer screening, surveillance

## Abstract

**Objective:**

Colonoscopy surveillance aims to reduce colorectal cancer (CRC) incidence after polypectomy. The 2020 UK guidelines recommend surveillance at 3 years for ‘high-risk’ patients with ≥2 premalignant polyps (PMPs), of which ≥1 is ‘advanced’ (serrated polyp (or adenoma) ≥10 mm or with (high-grade) dysplasia); ≥5 PMPs; or ≥1 non-pedunculated polyp ≥20 mm; ‘low-risk’ patients without these findings are instead encouraged to participate in population-based CRC screening. We examined the appropriateness of these risk classification criteria and recommendations.

**Design:**

Retrospective analysis of patients who underwent colonoscopy and polypectomy mostly between 2000 and 2010 at 17 UK hospitals, followed-up through 2017. We examined CRC incidence by baseline characteristics, risk group and number of surveillance visits using Cox regression, and compared incidence with that in the general population using standardised incidence ratios (SIRs).

**Results:**

Among 21 318 patients, 368 CRCs occurred during follow-up (median: 10.1 years). Baseline CRC risk factors included age ≥55 years, ≥2 PMPs, adenomas with tubulovillous/villous/unknown histology or high-grade dysplasia, proximal polyps and a baseline visit spanning 2–90 days. Compared with the general population, CRC incidence without surveillance was higher among those with adenomas with high-grade dysplasia (SIR 1.74, 95% CI 1.21 to 2.42) or ≥2 PMPs, of which ≥1 was advanced (1.39, 1.09 to 1.75). For low-risk (71%) and high-risk (29%) patients, SIRs without surveillance were 0.75 (95% CI 0.63 to 0.88) and 1.30 (1.03 to 1.62), respectively; for high-risk patients after first surveillance, the SIR was 1.22 (0.91 to 1.60).

**Conclusion:**

These guidelines accurately classify post-polypectomy patients into those at high risk, for whom one surveillance colonoscopy appears appropriate, and those at low risk who can be managed by non-invasive screening.

**Significance of this study**

**What is already known on this subject?**

- Post-polypectomy surveillance aims to prevent colorectal cancer (CRC), or detect it early, following the removal of premalignant polyps (PMPs).
- The UK, EU and US surveillance guidelines were updated in 2020 to incorporate new data on long-term CRC incidence and mortality.
- The new UK guidelines recommend that ‘high-risk’ patients with ≥2 PMPs, of which ≥1 is ‘advanced’ (adenoma ≥10 mm or with high-grade dysplasia; serrated polyp ≥10 mm or with dysplasia); ≥5 PMPs; or a single large (≥20 mm) non-pedunculated polyp undergo surveillance colonoscopy at 3 years. ‘Low-risk’ patients without these findings are encouraged to participate in their national CRC screening programme when invited rather than undergo surveillance.
- The accuracy of the classification criteria and the appropriateness of the surveillance recommendations in the new UK guidelines have not been investigated.

**Significance of this study**

**What are the new findings?**

- In our cohort of ~21 000 patients with polyps, only those who had an adenoma with high-grade dysplasia or ≥2 PMPs, of which ≥1 was advanced, remained at increased risk of CRC after polypectomy.
- Applying the risk classification criteria in the new UK guidelines, 71% and 29% of our cohort were classified as low risk and high risk, respectively.
- Compared with the general population, CRC incidence was 25% lower among low-risk patients and 30% higher among high-risk patients in the absence of surveillance.
- The excess risk in high-risk patients was reduced after one surveillance visit.

**How might it impact on clinical practice in the foreseeable future?**

- Healthcare professionals can be reassured that the new UK guidelines accurately identify patients at increased risk after polypectomy, and that a one-off surveillance colonoscopy is appropriate for these patients.
- The new UK guidelines will also help ensure that low-risk patients are not exposed to unnecessary surveillance procedures and are appropriately managed by population-based non-invasive CRC screening instead.

## Introduction

Colorectal cancer (CRC) can be prevented by removing premalignant polyps (PMPs), which include adenomatous and serrated polyps.^1^ However, as polyps can recur, some patients are recommended surveillance colonoscopy to prevent future CRC. National guidelines tailor surveillance strategies according to baseline polyp characteristics.^2–7^ Guidelines have largely been based on studies using surrogate endpoints for CRC, a method prone to overestimating risk, due to a lack of data on long-term post-polypectomy CRC outcomes. However, in 2020, the UK, EU and US post-polypectomy surveillance guidelines were revised to incorporate new data on long-term CRC incidence and mortality.^6–8^

The 2020 UK guidelines recommend surveillance at 3 years for patients with ≥2 PMPs, of which ≥1 is ‘advanced’ (adenoma ≥10 mm or with high-grade dysplasia; serrated polyp ≥10 mm or with dysplasia); ≥5 PMPs; or ≥1 large (≥20 mm) non-pedunculated PMP (LNPPMP).^6^ Patients without these findings are deemed at low risk and are encouraged to participate in their national CRC screening programme when invited rather than undergo surveillance. The 2020 EU and US guidelines use similar polyp characteristics to identify patients requiring surveillance (eg, PMPs ≥10 mm, high-grade dysplasia, ≥5 PMPs).^7^

Several studies informed these guideline revisions^9–17^; however, only one of these compared post-polypectomy CRC incidence without surveillance to that in the general population, which is essential in determining surveillance requirements. This was our previous study of 11 944 patients classified at baseline colonoscopy as ‘intermediate risk’ according to the 2002 UK surveillance guidelines.^2 9 10^ Our analyses identified baseline CRC risk factors (incomplete colonoscopies, poor bowel preparation, adenomas ≥20 mm, adenomas with high-grade dysplasia, proximal polyps) which discriminated patients remaining at increased risk after polypectomy and in need of surveillance from those not.^9 10^

The authors of the new UK guidelines highlighted the need for further studies assessing long-term post-polypectomy CRC outcomes. The present study examined post-polypectomy CRC incidence by baseline patient, procedural and polyp characteristics among ~21 300 patients over a median of 10.1 years and assessed the appropriateness of the risk classification criteria and surveillance recommendations in the new UK guidelines.^6^

## Methods

### Study design and participants

This retrospective cohort study used data from patients who underwent colonoscopy with polypectomy at 17 UK hospitals from 1984 to 2010 (mostly (87%) from 2000 to 2010). We previously used this cohort for our study of patients classified as ‘intermediate risk’ according to the 2002 UK guidelines,^2 9 10^ and a study examining all risk groups in these former guidelines (‘low risk’, ‘intermediate risk’, ‘high risk’).^18^ For the present study, we obtained additional follow-up data on cancers and deaths. We examined the whole cohort combined and performed a stratified analysis applying the risk classification criteria in the 2020 UK guidelines.^6^

Participating hospitals were required to have at least 6 years’ worth of electronically recorded endoscopy and pathology data for patients undergoing colonic examination prior to the study start (2006). We searched hospital endoscopy databases for patients with colonic examinations before 31 December 2010 and pathology databases for records describing colorectal lesions. We linked and pseudonymised endoscopy and pathology reports and entered them into a database (Oracle Corporation, Redwood City, California, USA). We assigned summary values for size, histology and location to lesions seen at >1 examination.^10^

Once we had identified patients with colonic examinations before 31 December 2010, we examined their records to identify the first adenoma diagnosis, which we defined as ‘baseline’. In some cases, >1 examination was required at baseline to completely examine the colon and remove all detected lesions; we grouped these examinations into the ‘baseline visit’. Baseline visits could extend over multiple days. We grouped colonic examinations occurring after the baseline visit into surveillance visits.^10^ We collected data on colonic examinations through 2016.

To be included, patients were required to have had a colonoscopy and ≥1 adenomas at baseline. We excluded patients with CRC or a bowel resection at or before baseline; inflammatory bowel disease or colitis; Lynch syndrome or family history of familial adenomatous polyposis; polyposis, juvenile polyps or hamartomatous polyps; colorectal carcinoma in situ (now described as high-grade dysplasia) reported in registry data >3 years before baseline, which we thought had the potential to progress to invasive carcinoma by baseline; an examination without a recorded date; or were missing information required for risk classification.

We additionally excluded patients whose baseline colonoscopy was suboptimal (incomplete or of unknown completeness, or with poor bowel preparation) so that our data reflect contemporary high-quality colonoscopy practice. Suboptimal baseline colonoscopies were associated with increased CRC risk in our previous studies of this cohort.^9 10 18^

Data on cancers and deaths were provided by the National Health Service (NHS) Central Register, National Services Scotland and NHS Digital through 2016 (Scotland) or 2017 (England). We compared the cancer data with the pathology data on the database and resolved duplicate and inconsistent records.

The primary outcome was incident adenocarcinoma of the colorectum. This included cancers with unspecified morphology if they were located between the caecum and rectum, but not if they were located around the anus; we assumed the former were adenocarcinomas, the latter squamous cell carcinomas. In-situ cancers were not included.

We excluded CRCs that we assumed had developed from incompletely excised baseline lesions (n=25); those found in the same/neighbouring colonic segment to an adenoma measuring ≥15 mm at baseline and seen at least twice within 5 years before the cancer diagnosis.^9 10 18^ We did this so that our data reflect current practice, considering the improvements in quality of endoscopic excision over the past decade.^19^ In a sensitivity analysis, we did not make this exclusion.

We classified patients into ‘low-risk’ and ‘high-risk’ groups based on the 2020 UK guidelines.^6^ High-risk patients were those with ≥2 PMPs, of which ≥1 was ‘advanced’ (adenoma ≥10 mm or with high-grade dysplasia; serrated polyp ≥10 mm or with dysplasia); ≥5 PMPs; or ≥1 LNPPMP. Patients without these findings were classified as low risk.

We did not create separate serrated polyp variables because serrated polyps were not consistently recorded or classified in the era of our data, and patients in our cohort with serrated polyps were a selected subgroup of patients with both adenomas and serrated polyps at baseline. However, we used any available serrated polyp data in our classification of risk (ie, in the count of PMPs and advanced PMPs). Our definition of serrated polyps included hyperplastic polyps and sessile-serrated lesions. In the 2020 UK guidelines, serrated polyps also include serrated adenomas and mixed hyperplastic-adenomatous polyps^6^; however, these would likely have been recorded as adenomas in the age of our data and so we included them as such.^9 10 18^

### Statistical analysis

We used χ^2^ tests to compare baseline characteristics among patients with and without surveillance visits, and among low-risk and high-risk patients.

We performed the following analyses for the whole cohort and both risk groups. We estimated long-term CRC incidence after polypectomy. Time-at-risk started from the latest examination at baseline. We censored time-to-event data at first CRC diagnosis, emigration, death or the date cancer registration data was considered complete. Exposure to successive surveillance visits started at the latest examination in each visit. We did not include visits at which CRC was diagnosed as surveillance visits because they offered no protection against CRC. We divided each patient’s follow-up time into three periods: without surveillance, censoring at any first surveillance; after first surveillance, censoring at any second surveillance; and after second surveillance, censoring at end of follow-up. For the whole cohort and low-risk group, we combined the last two periods in some analyses to estimate CRC incidence in the presence of ≥1 surveillance visits.

We examined effects of baseline characteristics and surveillance on CRC incidence using univariable and multivariable Cox proportional hazards models to estimate HRs with 95% CIs. Baseline characteristics of interest included sex, age, number and size of PMPs, adenoma histology and dysplasia, proximal polyps, year of baseline visit, length of baseline visit (in days) and family history of cancer/CRC. We identified independent CRC risk factors in the whole cohort in multivariable models using backward stepwise selection to retain variables with p values <0.05 in likelihood ratio tests. We included number of surveillance visits as a time-varying covariate. As we excluded patients with poor bowel preparation from this analysis, we do not present CRC incidence by bowel preparation quality because we previously showed that CRC incidence is similar among the remaining categories (‘excellent or good’, ‘satisfactory’, and ‘unknown’).^9^

We performed Kaplan-Meier analyses to show time to CRC diagnosis and estimate cumulative CRC incidence at 10 years with 95% CIs. We compared cumulative incidence curves using the log-rank test. We calculated standardised incidence ratios (SIRs) with exact Poisson 95% CIs, dividing the observed by the expected number of CRC cases. We estimated expected cases by multiplying sex-specific and 5-year age-group-specific person-years with the corresponding CRC incidence in the general population of England in 2007 (approximately the middle of the follow-up period).^20^ As the need for surveillance is determined by comparing CRC incidence without surveillance to that in the general population,^6^ our analysis of SIRs in the absence of surveillance was the main focus of our study.

We performed analyses in Stata/IC V.13.1.^21^ The study is registered (ISRCTN15213649). The protocol is available online.^22^

## Results

**Figure 1:**
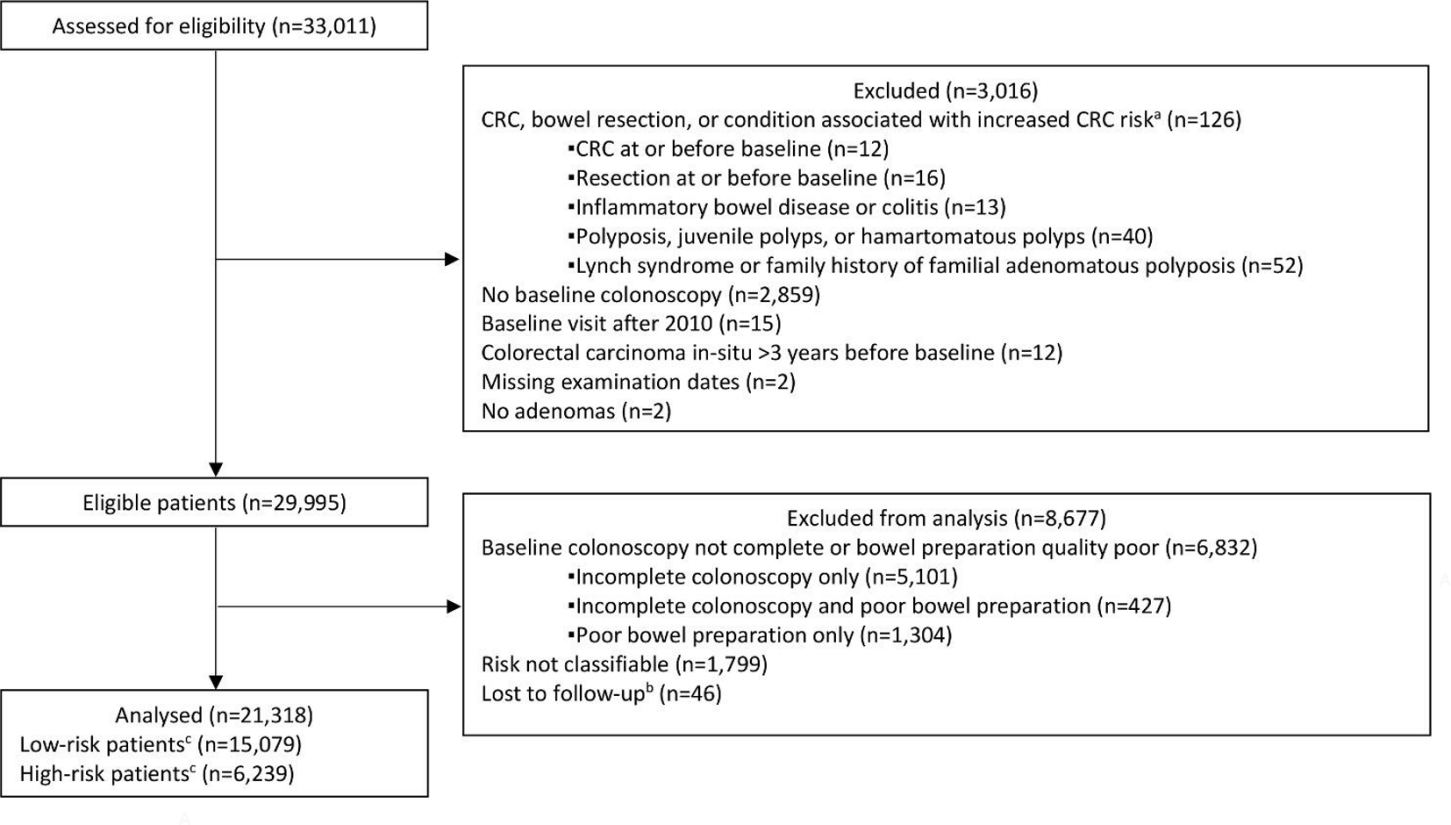
Study profile flow diagram. ^a^Not mutually exclusive. ^b^Reasons for lost to follow-up included having all examinations after emigrating (n=20); having no surveillance and being untraceable through national data sources (n=22); and having an unknown date of birth (n=4). ^c^High-risk patients were those with ≥2 premalignant polyps, of which ≥1 was advanced, ≥5 premalignant polyps or ≥1 large (≥20 mm) non-pedunculated premalignant polyp; low-risk patients had none of these findings. CRC, colorectal cancer.

The cohort included 33 011 patients. Of these, we excluded 126 with CRC or a bowel resection at or before baseline or a condition associated with increased CRC risk; 2859 without a baseline colonoscopy; 15 with a baseline visit after 2010; 12 with colorectal carcinoma in situ reported in registry data >3 years before baseline; 2 with missing examination dates; 2 with no adenomas; 1799 who were missing information needed for risk classification; 6832 whose baseline colonoscopy was not complete or bowel preparation quality was poor; and 46 who were lost to follow-up. This left 21 318 for analysis (figure 1).

In the whole cohort, the median age was 65 years (IQR 57–72), 42% were female and 54% attended ≥1 surveillance visits (table 1). The median time from baseline to first surveillance was 3.0 years (IQR 1.5–4.1). Patients attending surveillance (n=11 604) were younger than non-attenders (n=9714) and more likely to have had, at baseline, a greater number of PMPs, PMPs ≥10 mm, adenomas with tubulovillous/villous histology or high-grade dysplasia, proximal polyps, a baseline visit before 2005, a baseline visit spanning >1 day, a family history of cancer/CRC or missing data (online supplemental table 1).

10.1136/gutjnl-2020-323411.supp1 Supplementary data

**Table 1:** Long-term incidence of colorectal cancer by number of surveillance visits and baseline characteristics (n=21 318)

	n	%	No of person-years	No of CRCs	Incidence rate per 100 000 person-years (95% CI)	Univariable HR (95% CI)	P value*	Multivariable HR (95% CI)†	P value*
Total	21 318	100	210 814	368	175 (158 to 193)				
No of surveillance visits‡							<0.001		<0.001
0	9714	45.6	116 248	214	184 (161 to 210)	1		1	
1	5903	27.7	56 923	96	169 (138 to 206)	0.72 (0.56 to 0.92)		0.65 (0.50 to 0.84)	
2	3515	16.5	25 058	32	128 (90 to 181)	0.49 (0.33 to 0.71)		0.43 (0.29 to 0.63)	
≥3	2186	10.3	12 586	26	207 (141 to 303)	0.66 (0.43 to 1.03)		0.54 (0.35 to 0.85)	
Sex							0.93		0.90
Women	9022	42.3	92 173	161	175 (150 to 204)	1		1	
Men	12 296	57.7	118 641	207	174 (152 to 200)	1.01 (0.82 to 1.24)		1.01 (0.82 to 1.25)	
Age at baseline, years							<0.001		<0.001
<55	4298	20.2	51 463	36	70 (50 to 97)	1		1	
55–64	5956	27.9	64 938	77	119 (95 to 148)	1.75 (1.18 to 2.60)		1.61 (1.08 to 2.40)	
65–74	6894	32.3	65 186	158	242 (207 to 283)	3.78 (2.63 to 5.44)		3.27 (2.27 to 4.72)	
≥75	4170	19.6	29 228	97	332 (272 to 405)	5.66 (3.84 to 8.34)		4.31 (2.91 to 6.38)	
No of PMPs							<0.001		0.003
1	12 231	57.4	124 117	163	131 (113 to 153)	1		1	
2	4714	22.1	45 601	100	219 (180 to 267)	1.70 (1.33 to 2.18)		1.36 (1.07 to 1.71)	
3	2035	9.6	19 482	41	210 (155 to 286)	1.63 (1.16 to 2.30)			
4	951	4.5	8856	23	260 (173 to 391)	2.02 (1.31 to 3.13)			
≥5	1387	6.5	12 760	41	321 (237 to 436)	2.53 (1.79 to 3.56)		1.82 (1.25 to 2.66)	
PMP size, mm§							<0.001		0.46
<10	11 553	54.2	116 281	166	143 (123 to 166)	1		1	
10–19	6081	28.5	59 382	109	184 (152 to 221)	1.29 (1.01 to 1.64)		1.06 (0.81 to 1.38)	
≥20	3625	17.0	34 544	92	266 (217 to 327)	1.87 (1.45 to 2.42)		1.28 (0.93 to 1.76)	
Unknown	59	0.3	607	1	165 (23 to 1169)	1.11 (0.16 to 7.92)		0.69 (0.10 to 5.03)	
Adenoma histology¶							<0.001		<0.001
Tubular	12 786	60.0	127 882	171	134 (115 to 155)	1		1	
Tubulovillous	6480	30.4	62 187	137	220 (186 to 260)	1.66 (1.33 to 2.08)		1.42 (1.12 to 1.80)	
Villous	1045	4.9	9958	31	311 (219 to 443)	2.35 (1.61 to 3.45)		1.60 (1.07 to 2.40)	
Unknown	1007	4.7	10 787	29	269 (187 to 387)	1.94 (1.31 to 2.88)		2.06 (1.37 to 3.11)	
Adenoma dysplasia**							<0.001		0.03
Low grade	18 592	87.2	183 696	290	158 (141 to 177)	1		1	
High grade	2148	10.1	19 913	63	316 (247 to 405)	2.03 (1.54 to 2.66)		1.51 (1.12 to 2.02)	
Unknown	578	2.7	7206	15	208 (125 to 345)	1.22 (0.72 to 2.06)		1.22 (0.71 to 2.11)	
Proximal polyps††							<0.001		<0.001
No	11 566	54.3	118 513	152	128 (109 to 150)	1		1	
Yes	9752	45.8	92 301	216	234 (205 to 267)	1.86 (1.51 to 2.29)		1.63 (1.30 to 2.05)	
Year of baseline visit							0.81		0.34
1984–1999	2057	9.7	28 319	60	212 (165 to 273)	1		1	
2000–2004	6651	31.2	74 494	137	184 (156 to 217)	0.96 (0.69 to 1.34)		0.89 (0.64 to 1.23)	
2005–2010	12 610	59.2	108 001	171	158 (136 to 184)	0.91 (0.65 to 1.27)		0.78 (0.56 to 1.10)	
Length of baseline visit, days							<0.001		0.04
1	14 223	66.7	140 884	208	148 (129 to 169)	1		1	
2–90	3035	14.2	29 429	70	238 (188 to 301)	1.63 (1.24 to 2.13)		1.50 (1.13 to 1.99)	
91–183	2085	9.8	21 071	43	204 (151 to 275)	1.38 (0.99 to 1.92)		1.21 (0.86 to 1.71)	
≥184	1975	9.3	19 430	47	242 (182 to 322)	1.63 (1.19 to 2.24)		1.30 (0.92 to 1.82)	
Family history of cancer/CRC‡‡							0.22		0.10
No	19 730	92.6	191 764	340	177 (159 to 197)	1		1	
Yes	1588	7.5	19 051	28	147 (101 to 213)	0.79 (0.54 to 1.16)		1.42 (0.95 to 2.11)	

*P values were calculated with the likelihood ratio test.

†The final multivariable model contained number of surveillance visits, age, number of PMPs, adenoma histology, adenoma dysplasia, proximal polyps and length of baseline visit. For these variables, the multivariable HRs were from the final multivariable model and the p values were for inclusion of the variable in the model. For the remaining variables, the multivariable HRs were for if the variable was added as an additional variable to the final multivariable model.

‡Number of surveillance visits was included as a time-varying covariate, meaning that patients who had surveillance contributed person-years to more than a single category of number of surveillance visits.

§PMP size was defined according to the largest PMP seen at baseline.

¶Adenoma histology was defined according to the greatest degree of villousness seen at baseline.

**Adenoma dysplasia was defined according to the highest grade of dysplasia seen at baseline.

††Proximal polyps were defined as those proximal to the descending colon.

‡‡Family history of cancer/CRC was defined as ‘family history of cancer or CRC reported at an examination before or during visit’. Of cases with a ‘family history of cancer’, 72% were from a specialist hospital for colorectal diseases and so we assumed these cases had a family history of CRC.

CRC, colorectal cancer; mm, millimetre; PMP, premalignant polyp.

Over a median follow-up of 10.1 years (IQR 7.5–12.7), 368 CRCs were diagnosed, giving an incidence rate of 175 per 100 000 person-years (95% CI 158 to 193). Attendance at ≥1 surveillance visits was independently associated with reduced CRC incidence, while age ≥55 years and having ≥2 PMPs, an adenoma with tubulovillous/villous/unknown histology or high-grade dysplasia, proximal polyps or a baseline visit spanning 2–90 days were independently associated with increased CRC incidence (table 1).

**Figure 2:**
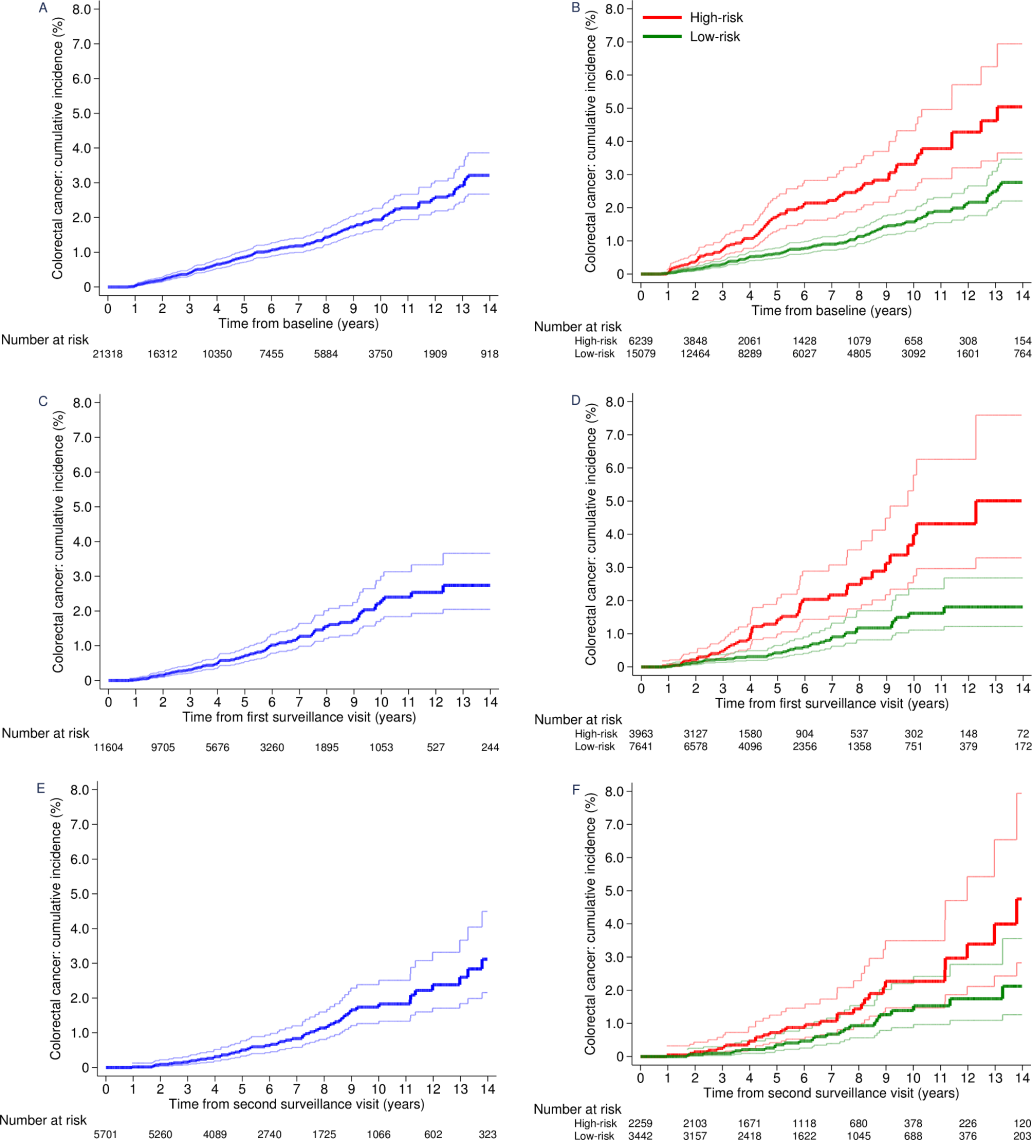
Cumulative incidence of colorectal cancer by time from baseline, first surveillance and second surveillance. Cumulative incidence of colorectal cancer without surveillance (censoring at any first surveillance visit) for the whole cohort (A) and for low-risk and high-risk patients (B). Cumulative incidence of colorectal cancer after first surveillance (censoring at any second surveillance visit) for the whole cohort (C) and for low-risk and high-risk patients (D). Cumulative incidence of colorectal cancer after second surveillance (censoring at end of follow-up) for the whole cohort (E) and for low-risk and high-risk patients (F). 95% CIs are shown for each curve. High-risk patients were those with ≥2 premalignant polyps, of which ≥1 was advanced, ≥5 premalignant polyps or ≥1 large (≥20 mm) non-pedunculated premalignant polyp; low-risk patients had none of these findings.

Without surveillance, in the whole cohort, cumulative CRC incidence at 10 years was 1.9% (95% CI 1.7% to 2.3%) (table 2; figure 2A) and CRC incidence was similar to that in the general population (SIR 0.88, 95% CI 0.77 to 1.01) (table 2). Incidence of CRC was lower than in the general population among men (SIR 0.78, 95% CI 0.64 to 0.93), patients aged 55–64 years (0.71, 0.50 to 0.98), and patients with a single PMP (0.71, 0.58 to 0.86), PMPs <10 mm (0.77, 0.64 to 0.93), adenomas with tubular histology (0.77, 0.63 to 0.92), adenomas with low-grade dysplasia (0.80, 0.69 to 0.93) or no proximal polyps (0.66, 0.53 to 0.82) at baseline. In contrast, CRC incidence without surveillance was higher among patients with adenomas with high-grade dysplasia (SIR 1.74, 95% CI 1.21 to 2.42) or ≥2 PMPs, of which ≥1 was advanced (1.39, 1.09 to 1.75) than in the general population (table 2).

**Table 2:** Cumulative incidence of colorectal cancer and age-sex-standardised incidence ratios in the whole cohort (n=21 318)

	n	%	No of person-years	No of CRCs	Incidence rate per 100 000 person-years (95% CI)	At 10 years		P value†	Standardisation	
						No of CRCs	Cumulative incidence (95% CI)*		No of expected CRCs‡	SIR (95% CI)
**After baseline (without surveillance, censored at any first surveillance visit**)										
Total	21 318	100	116 248	214	184 (161 to 210)	183	1.9% (1.7 to 2.3)		242	0.88 (0.77 to 1.01)
Sex								0.50		
Women	9022	42	52 431	93	177 (145 to 217)	74	1.7% (1.3 to 2.2)		87	1.08 (0.87 to 1.32)
Men	12 296	58	63 816	121	190 (159 to 227)	109	2.1% (1.7 to 2.6)		156	0.78 (0.64 to 0.93)
Age at baseline, years								<0.001		
<55	4298	20	26 718	12	45 (26 to 79)	9	0.4% (0.2 to 0.8)		13	0.93 (0.48 to 1.63)
55–64	5956	28	32 358	36	111 (80 to 154)	30	1.3% (0.9 to 2.0)		51	0.71 (0.50 to 0.98)
65–74	6894	32	35 831	94	262 (214 to 321)	81	2.6% (2.1 to 3.3)		100	0.94 (0.76 to 1.15)
≥75	4170	20	21 341	72	337 (268 to 425)	63	3.6% (2.7 to 4.7)		79	0.92 (0.72 to 1.15)
No of PMPs								<0.001		
1	12 231	57	72 860	102	140 (115 to 170)	82	1.4% (1.1 to 1.8)		144	0.71 (0.58 to 0.86)
2	4714	22	24 974	59	236 (183 to 305)	51	2.4% (1.8 to 3.2)		56	1.06 (0.81 to 1.37)
3	2035	10	9612	22	229 (151 to 348)	20	2.9% (1.8 to 4.6)		22	1.00 (0.62 to 1.51)
4	951	4	3971	14	353 (209 to 595)	14	5.4% (3.0 to 9.7)		9	1.55 (0.84 to 2.59)
≥5	1387	7	4830	17	352 (219 to 566)	16	3.7% (2.0 to 6.5)		11	1.56 (0.91 to 2.49)
PMP size, mm§								0.001		
<10	11 553	54	72 061	112	155 (129 to 187)	95	1.6% (1.3 to 2.0)		145	0.77 (0.64 to 0.93)
10–19	6081	29	29 408	62	211 (164 to 270)	52	2.2% (1.6 to 3.1)		64	0.97 (0.75 to 1.25)
≥20	3625	17	14 553	39	268 (196 to 367)	35	3.0% (2.0 to 4.4)		33	1.18 (0.84 to 1.61)
Adenoma histology¶								0.002		
Tubular	12 786	60	75 483	117	155 (129 to 186)	100	1.6% (1.3 to 2.0)		153	0.77 (0.63 to 0.92)
Tubulovillous	6480	30	30 698	68	222 (175 to 281)	58	2.4% (1.8 to 3.2)		68	1.00 (0.78 to 1.27)
Villous	1045	5	4505	14	311 (184 to 525)	13	3.2% (1.7 to 5.9)		11	1.29 (0.70 to 2.16)
Unknown	1007	5	5562	15	270 (163 to 447)	12	3.1% (1.7 to 5.8)		10	1.45 (0.81 to 2.40)
Adenoma dysplasia**								<0.001		
Low grade	18 592	87	104 400	173	166 (143 to 192)	145	1.7% (1.4 to 2.0)		215	0.80 (0.69 to 0.93)
High grade	2148	10	8373	35	418 (300 to 582)	33	5.2% (3.6 to 7.7)		20	1.74 (1.21 to 2.42)
Unknown	578	3	3475	6	173 (78 to 384)	5	2.2% (0.8 to 5.8)		7	0.87 (0.32 to 1.89)
Proximal polyps††								<0.001		
No	11 566	54	67 073	88	131 (106 to 162)	77	1.5% (1.2 to 1.9)		133	0.66 (0.53 to 0.82)
Yes	9752	46	49 174	126	256 (215 to 305)	106	2.5% (2.0 to 3.1)		110	1.15 (0.96 to 1.37)
No of APMPs and PMPs								<0.001		
No APMPs, 1 PMP	7506	35	49 423	66	134 (105 to 170)	53	1.3% (1.0 to 1.8)		96	0.69 (0.53 to 0.88)
No APMPs, 2–4 PMPs	3346	16	19 581	38	194 (141 to 267)	34	2.2% (1.6 to 3.2)		43	0.89 (0.63 to 1.22)
No APMPs, ≥5 PMPs	461	2	1991	3	151 (49 to 467)	3	1.4% (0.4 to 4.5)		4	0.73 (0.15 to 2.14)
1 APMP, no other PMPs	4725	22	23 437	36	154 (111 to 213)	29	1.6% (1.1 to 2.4)		49	0.74 (0.52 to 1.02)
≥1 APMP, ≥2 total PMPs	5280	25	21 815	71	325 (258 to 411)	64	3.6% (2.7 to 4.8)		51	1.39 (1.09 to 1.75)
**After first surveillance (with one or more surveillance visits, censored at end of follow-up**)										
Total	11 604	100	94 567	154	163 (139 to 191)	122	1.6% (1.4 to 2.0)		213	0.72 (0.61 to 0.85)
Sex								0.66		
Women	4804	41	39 742	68	171 (135 to 217)	56	1.9% (1.4 to 2.5)		67	1.02 (0.79 to 1.29)
Men	6800	59	54 825	86	157 (127 to 194)	66	1.5% (1.1 to 1.9)		146	0.59 (0.47 to 0.73)
Age at baseline, years								<0.001		
<55	2702	23	24 746	24	97 (65 to 145)	19	0.9% (0.6 to 1.4)		19	1.25 (0.80 to 1.86)
55–64	3799	33	32 580	41	126 (93 to 171)	30	1.2% (0.8 to 1.8)		69	0.60 (0.43 to 0.81)
65–74	3780	33	29 354	64	218 (171 to 279)	51	2.3% (1.7 to 3.1)		95	0.68 (0.52 to 0.86)
≥75	1323	11	7887	25	317 (214 to 469)	22	3.7% (2.3 to 6.0)		30	0.83 (0.53 to 1.22)
No of PMPs								<0.001		
1	6188	53	51 257	61	119 (93 to 153)	51	1.3% (1.0 to 1.7)		108	0.57 (0.43 to 0.73)
2	2617	23	20 626	41	199 (146 to 270)	28	1.6% (1.1 to 2.4)		48	0.85 (0.61 to 1.16)
3	1225	11	9870	19	193 (123 to 302)	15	1.7% (1.0 to 2.9)		24	0.79 (0.48 to 1.23)
4	596	5	4884	9	184 (96 to 354)	6	1.2% (0.5 to 2.7)		12	0.73 (0.33 to 1.38)
≥5	978	8	7930	24	303 (203 to 452)	22	4.0% (2.5 to 6.3)		21	1.17 (0.75 to 1.74)
PMP size, mm§								<0.001		
<10	5608	48	44 221	54	122 (94 to 159)	44	1.3% (0.9 to 1.7)		93	0.58 (0.43 to 0.75)
10–19	3591	31	29 974	47	157 (118 to 209)	39	1.5% (1.1 to 2.1)		70	0.67 (0.50 to 0.90)
≥20	2366	20	19 991	53	265 (203 to 347)	39	2.7% (1.9 to 3.7)		48	1.10 (0.82 to 1.44)
Adenoma histology¶								<0.001		
Tubular	6526	56	52 399	54	103 (79 to 135)	42	0.9% (0.7 to 1.3)		114	0.48 (0.36 to 0.62)
Tubulovillous	3849	33	31 489	69	219 (173 to 277)	57	2.4% (1.8 to 3.2)		74	0.94 (0.73 to 1.19)
Villous	660	6	5453	17	312 (194 to 501)	13	3.0% (1.7 to 5.5)		14	1.21 (0.70 to 1.93)
Unknown	569	5	5225	14	268 (159 to 452)	10	2.6% (1.4 to 5.0)		11	1.23 (0.67 to 2.06)
Adenoma dysplasia**								0.05		
Low grade	9857	85	79 296	117	148 (123 to 177)	92	1.5% (1.2 to 1.8)		175	0.67 (0.55 to 0.80)
High grade	1389	12	11 539	28	243 (168 to 351)	25	2.7% (1.8 to 4.1)		29	0.95 (0.63 to 1.38)
Unknown	358	3	3731	9	241 (126 to 464)	5	1.8% (0.7 to 4.3)		8	1.10 (0.50 to 2.09)
Proximal polyps††								<0.001		
No	6195	53	51 440	64	124 (97 to 159)	50	1.2% (0.9 to 1.7)		109	0.59 (0.45 to 0.75)
Yes	5409	47	43 126	90	209 (170 to 257)	72	2.1% (1.7 to 2.7)		103	0.87 (0.70 to 1.07)
No of APMPs and PMPs								<0.001		
No APMPs, 1 PMP	3402	29	26 997	27	100 (69 to 146)	23	1.1% (0.7 to 1.7)		54	0.50 (0.33 to 0.73)
No APMPs, 2–4 PMPs	1748	15	13 362	17	127 (79 to 205)	11	1.0% (0.5 to 1.9)		30	0.57 (0.33 to 0.91)
No APMPs, ≥5 PMPs	310	3	2566	6	234 (105 to 520)	6	3.1% (1.4 to 7.2)		6	0.95 (0.35 to 2.06)
1 APMP, no other PMPs	2786	24	24 259	34	140 (100 to 196)	28	1.5% (1.0 to 2.2)		54	0.64 (0.44 to 0.89)
≥1 APMP, ≥2 total PMPs	3358	29	27 382	70	256 (202 to 323)	54	2.4% (1.8 to 3.3)		69	1.02 (0.79 to 1.29)

*Cumulative CRC incidence was estimated using the Kaplan-Meier method.

†P values were calculated with the log-rank test to compare cumulative CRC incidence among each category of the specified variable.

‡Numbers of expected CRCs were calculated by multiplying the 5-year age-group and sex-specific observed person-years by the corresponding CRC incidence rates in the general population of England in 2007.

§PMP size was defined according to the largest PMP seen at baseline. Patients with PMPs of unknown size are not included in the table; in the analyses without surveillance, there were 59 such patients, of whom one was diagnosed with CRC; and in the analyses with one or more surveillance visits, there were 39 such patients with no CRC cases.

¶Adenoma histology was defined according to the greatest degree of villousness seen at baseline.

**Adenoma dysplasia was defined according to the highest grade of dysplasia seen at baseline.

††Proximal polyps were defined as those proximal to the descending colon.

APMP, advanced PMP; CRC, colorectal cancer; PMP, premalignant polyp; SIR, standardised incidence ratio.

In the presence of ≥1 surveillance visits, cumulative CRC incidence in the whole cohort was 1.6% (95% CI 1.4% to 2.0%) at 10 years (table 2; figure 2C). Incidence of CRC among all patients was lower than in the general population (SIR 0.72, 95% CI 0.61 to 0.85) and no longer significantly higher among those with adenomas with high-grade dysplasia (SIR 0.95, 95% CI 0.63 to 1.38) or ≥2 PMPs, of which ≥1 was advanced (1.02, 0.79 to 1.29) (table 2).

### Low-risk and high-risk groups

We then classified patients into low-risk (n=15 079, 71%) and high-risk (n=6239, 29%) groups (tables 3–5).^6^

**Table 3:** Effects of surveillance on colorectal cancer incidence by number of surveillance visits and risk group

	n	%	No of person-years	No of CRCs	Incidence rate per 100 000 person-years (95% CI)	Effect of surveillance on CRC incidence*			
						Univariable HR (95% CI)	P value†	Multivariable HR (95% CI)‡	P value†
Low-risk patients**§**							<0.001		0.001
0 visit	7438	49.3	90 451	136	150 (127 to 178)	1		1	
1 visit	4199	27.8	39 392	44	112 (83 to 150)	0.57 (0.40 to 0.81)		0.58 (0.41 to 0.83)	
≥2 visits	3442	22.8	22 654	26	115 (78 to 169)	0.48 (0.30 to 0.75)		0.53 (0.33 to 0.83)	
Total	15 079	70.7	152 497	206	135 (118 to 155)				
High-risk patients**§**							<0.001		0.002
0 visit	2276	36.5	25 796	78	302 (242 to 377)	1		1	
1 visit	1704	27.3	17 531	52	297 (226 to 389)	0.73 (0.51 to 1.05)		0.71 (0.49 to 1.03)	
≥2 visits	2259	36.2	14 990	32	213 (151 to 302)	0.42 (0.27 to 0.66)		0.44 (0.28 to 0.70)	
Total	6239	29.3	58 318	162	278 (238 to 324)				

*Number of surveillance visits was included as a time-varying covariate, meaning that patients who had surveillance contributed person-years to more than a single category of number of surveillance visits.

†P values were calculated with the likelihood ratio test.

‡Multivariable HR adjusted for age, number of premalignant polyps, adenoma histology, adenoma dysplasia, proximal polyps and length of baseline visit, the characteristics independently associated with CRC incidence in the whole cohort.

§High-risk patients were those with ≥2 premalignant polyps, of which ≥1 was advanced, ≥5 premalignant polyps, or ≥1 large (≥20 mm) non-pedunculated premalignant polyp; low-risk patients had none of these findings.

CRC, colorectal cancer.

**Table 4:** Cumulative incidence of colorectal cancer and age-sex-standardised incidence ratios in low-risk patients (n=15 079)

	n	%	No of person-years	No of CRCs	Incidence rate per 100 000 person-years (95% CI)	At 10 years		P value†	Standardisation	
						No of CRCs	Cumulative incidence (95% CI)*		No of expected CRCs‡	SIR (95% CI)
**After baseline (without surveillance, censored at any first surveillance visit)**										
Total	15 079	100	90 451	136	150 (127 to 178)	113	1.6% (1.3 to 1.9)		182	0.75 (0.63 to 0.88)
Sex								0.43		
Women	6796	45	42 473	60	141 (110 to 182)	45	1.3% (1.0 to 1.8)		68	0.88 (0.67 to 1.13)
Men	8283	55	47 978	76	158 (127 to 198)	68	1.8% (1.4 to 2.4)		114	0.67 (0.52 to 0.83)
Age at baseline, years								<0.001		
<55	3469	23	22 734	7	31 (15 to 65)	4	0.2% (0.1 to 0.6)		11	0.66 (0.26 to 1.35)
55−64	4193	28	25 273	24	95 (64 to 142)	20	1.1% (0.7 to 1.7)		40	0.61 (0.39 to 0.90)
65−74	4589	30	26 926	64	238 (186 to 304)	53	2.3% (1.8 to 3.1)		75	0.85 (0.66 to 1.09)
≥75	2828	19	15 518	41	264 (195 to 359)	36	3.0% (2.1 to 4.4)		57	0.72 (0.52 to 0.98)
No of PMPs								0.13		
1	11 733	78	70 870	98	138 (113 to 169)	79	1.4% (1.1 to 1.8)		140	0.70 (0.57 to 0.86)
2	2184	14	13 337	24	180 (121 to 268)	20	1.8% (1.1 to 2.9)		29	0.83 (0.53 to 1.24)
3	827	5	4 645	9	194 (101 to 372)	9	2.9% (1.5 to 5.5)		10	0.86 (0.39 to 1.64)
4	335	2	1 600	5	313 (130 to 751)	5	4.7% (1.7 to 12.9)		3	1.46 (0.47 to 3.40)
PMP size, mm§								0.09		
<10	10 985	73	69 586	105	151 (125 to 183)	88	1.6% (1.3 to 2.0)		140	0.75 (0.61 to 0.91)
10−19	2981	20	15 651	26	166 (113 to 244)	20	1.7% (1.0 to 2.8)		32	0.80 (0.53 to 1.18)
≥20	1086	7	5 102	4	78 (29 to 209)	4	1.1% (0.4 to 3.4)		10	0.40 (0.11 to 1.03)
Adenoma histology¶								0.22		
Tubular	10 376	69	64 774	88	136 (110 to 167)	76	1.4% (1.1 to 1.8)		129	0.68 (0.55 to 0.84)
Tubulovillous	3517	23	18 944	34	179 (128 to 251)	26	1.9% (1.3 to 3.0)		40	0.85 (0.59 to 1.19)
Villous	359	2	1 853	3	162 (52 to 502)	2	1.1% (0.2 to 4.5)		4	0.72 (0.15 to 2.10)
Unknown	827	5	4 880	11	225 (125 to 407)	9	2.7% (1.4 to 5.5)		9	1.23 (0.61 to 2.20)
Adenoma dysplasia**								0.79		
Low-grade	13 888	92	84 243	125	148 (125 to 177)	103	1.5% (1.3 to 1.9)		169	0.74 (0.62 to 0.88)
High-grade	740	5	3 321	6	181 (81 to 402)	6	2.2% (0.9 to 5.5)		7	0.81 (0.30 to 1.77)
Unknown	451	3	2 887	5	173 (72 to 416)	4	1.7% (0.6 to 5.2)		6	0.86 (0.28 to 2.00)
Proximal polyps††								<0.001		
No	9091	60	55 867	63	113 (88 to 144)	54	1.3% (1.0 to 1.8)		108	0.59 (0.45 to 0.75)
Yes	5988	40	34 585	73	211 (168 to 266)	59	1.9% (1.5 to 2.5)		75	0.98 (0.77 to 1.23)
**After first surveillance (with one or more surveillance visits, censored at end of follow-up)**										
Total	7641	100	62 045	70	113 (89 to 143)	55	1.1% (0.9 to 1.5)		131	0.54 (0.42 to 0.68)
Sex								0.09		
Women	3437	45	28 298	39	138 (101 to 189)	32	1.6% (1.1 to 2.3)		46	0.85 (0.60 to 1.16)
Men	4204	55	33 747	31	92 (65 to 131)	23	0.8% (0.5 to 1.2)		85	0.37 (0.25 to 0.52)
Age at baseline, years								0.007		
<55	2086	27	18 864	13	69 (40 to 119)	10	0.6% (0.3 to 1.2)		14	0.92 (0.49 to 1.57)
55−64	2500	33	21 251	22	104 (68 to 157)	15	0.9% (0.5 to 1.6)		44	0.50 (0.31 to 0.76)
65−74	2251	29	17 221	25	145 (98 to 215)	21	1.7% (1.1 to 2.7)		55	0.46 (0.29 to 0.67)
≥75	804	11	4710	10	212 (114 to 395)	9	2.4% (1.1 to 5.1)		18	0.56 (0.27 to 1.03)
No of PMPs								0.89		
1	5893	77	48 683	53	109 (83 to 143)	44	1.2% (0.9 to 1.6)		101	0.53 (0.39 to 0.69)
2	1096	14	8396	11	131 (73 to 237)	7	1.0% (0.5 to 2.3)		18	0.60 (0.30 to 1.07)
3	458	6	3464	4	115 (43 to 308)	3	1.0% (0.3 to 3.3)		8	0.50 (0.14 to 1.29)
4	194	3	1502	2	133 (33 to 532)	1	0.7% (0.1 to 5.0)		4	0.54 (0.07 to 1.96)
PMP size, mm§								0.43		
<10	5233	68	41 134	45	109 (82 to 147)	35	1.1% (0.8 to 1.6)		86	0.52 (0.38 to 0.70)
10−19	1674	22	14 519	14	96 (57 to 163)	11	1.0% (0.5 to 1.8)		32	0.44 (0.24 to 0.74)
≥20	717	9	6230	11	177 (98 to 319)	9	1.9% (1.0 to 3.7)		13	0.85 (0.42 to 1.51)
Adenoma histology¶								0.02		
Tubular	5016	66	39 874	33	83 (59 to 116)	24	0.7% (0.5 to 1.1)		83	0.40 (0.27 to 0.56)
Tubulovillous	1956	26	16 197	26	161 (109 to 236)	22	1.8% (1.1 to 2.7)		35	0.75 (0.49 to 1.09)
Villous	217	3	1860	2	108 (27 to 430)	1	0.7% (0.1 to 5.0)		4	0.45 (0.05 to 1.63)
Unknown	452	6	4115	9	219 (114 to 420)	8	2.7% (1.3 to 5.4)		9	1.03 (0.47 to 1.95)
Adenoma dysplasia**								0.71		
Low-grade	6912	90	55 214	63	114 (89 to 146)	49	1.1% (0.8 to 1.5)		116	0.55 (0.42 to 0.70)
High-grade	462	6	4059	3	74 (24 to 229)	3	0.9% (0.3 to 2.9)		9	0.32 (0.07 to 0.92)
Unknown	267	3	2772	4	144 (54 to 384)	3	1.6% (0.5 to 4.9)		6	0.70 (0.19 to 1.78)
Proximal polyps††								0.23		
No	4649	61	38 524	39	101 (74 to 139)	31	1.1% (0.7 to 1.5)		78	0.50 (0.36 to 0.68)
Yes	2992	39	23 521	31	132 (93 to 187)	24	1.2% (0.8 to 1.9)		53	0.59 (0.40 to 0.83)

Low-risk patients were those without any of the following: ≥2 PMPs, of which ≥1 was advanced, ≥5 PMPs or ≥1 large (≥20 mm) non-pedunculated PMP.

*Cumulative CRC incidence was estimated using the Kaplan-Meier method.

†P values were calculated with the log-rank test to compare cumulative CRC incidence among each category of the specified variable.

‡Numbers of expected CRCs were calculated by multiplying the 5-year age-group and sex-specific observed person-years by the corresponding CRC incidence rates in the general population of England in 2007.

§PMP size was defined according to the largest PMP seen at baseline. Patients with PMPs of unknown size are not included in the table; in the analyses without surveillance, there were 27 such patients, of whom one was diagnosed with CRC; and in the analyses with one or more surveillance visits, there were 17 such patients with no CRC cases.

¶Adenoma histology was defined according to the greatest degree of villousness seen at baseline.

**Adenoma dysplasia was defined according to the highest grade of dysplasia seen at baseline.

††Proximal polyps were defined as those proximal to the descending colon.

CRC, colorectal cancer; PMP, premalignant polyp; SIR, standardised incidence ratio.

**Table 5:** Cumulative incidence of colorectal cancer and age-sex-standardised incidence ratios in high-risk patients (n=6239)

	n	%	No of person-years	No of CRCs	Incidence rate per 100 000 person-years (95% CI)	At 10 years		P value†	Standardisation	
						No of CRCs	Cumulative incidence (95% CI)*		No of expected CRCs‡	SIR (95% CI)
**After baseline (without surveillance, censored at any first surveillance visit**)										
Total	6239	100	25 796	78	302 (242 to 377)	70	3.3% (2.5 to 4.3)		60	1.30 (1.03 to 1.62)
Sex								0.60		
Women	2226	36	9958	33	331 (236 to 466)	29	3.5% (2.3 to 5.3)		18	1.79 (1.23 to 2.51)
Men	4013	64	15 839	45	284 (212 to 381)	41	3.2% (2.2 to 4.5)		42	1.08 (0.79 to 1.45)
Age at baseline, years								<0.001		
<55	829	13	3983	5	126 (52 to 302)	5	1.4% (0.6 to 3.6)		2	2.27 (0.74 to 5.29)
55–64	1763	28	7085	12	169 (96 to 298)	10	2.6% (1.3 to 5.2)		11	1.08 (0.56 to 1.89)
65–74	2305	37	8905	30	337 (236 to 482)	28	3.4% (2.2 to 5.3)		25	1.20 (0.81 to 1.71)
≥75	1342	22	5823	31	532 (374 to 757)	27	5.2% (3.4 to 7.9)		22	1.44 (0.97 to 2.04)
No of PMPs								0.70		
1	498	8	1990	4	201 (75 to 536)	3	1.9% (0.6 to 6.2)		5	0.83 (0.23 to 2.13)
2	2530	41	11 638	35	301 (216 to 419)	31	3.1% (2.1 to 4.6)		27	1.30 (0.90 to 1.81)
3	1208	19	4967	13	262 (152 to 451)	11	2.9% (1.4 to 5.7)		12	1.12 (0.59 to 1.91)
4	616	10	2371	9	380 (197 to 729)	9	6.0% (2.9 to 12.2)		6	1.60 (0.73 to 3.04)
≥5	1387	22	4830	17	352 (219 to 566)	16	3.7% (2.0 to 6.5)		11	1.56 (0.91 to 2.49)
PMP size, mm§								0.35		
<10	568	9	2475	7	283 (135 to 593)	7	3.2% (1.4 to 7.3)		5	1.32 (0.53 to 2.72)
10–19	3100	50	13 757	36	262 (189 to 363)	32	2.9% (2.0 to 4.3)		31	1.15 (0.81 to 1.59)
≥20	2539	41	9451	35	370 (266 to 516)	31	4.0% (2.6 to 6.0)		23	1.52 (1.06 to 2.11)
Adenoma histology¶								0.31		
Tubular	2410	39	10 709	29	271 (188 to 390)	24	3.1% (2.0 to 4.7)		24	1.21 (0.81 to 1.74)
Tubulovillous	2963	47	11 753	34	289 (207 to 405)	32	3.0% (2.0 to 4.5)		28	1.21 (0.84 to 1.69)
Villous	686	11	2652	11	415 (230 to 749)	11	4.8% (2.3 to 9.8)		7	1.64 (0.82 to 2.94)
Unknown	180	3	682	4	587 (220 to 1563)	3	7.0% (1.6 to 27.9)		1	2.96 (0.81 to 7.57)
Adenoma dysplasia**								<0.001		
Low grade	4704	75	20 157	48	238 (179 to 316)	42	2.3% (1.6 to 3.2)		46	1.04 (0.77 to 1.38)
High grade	1408	23	5052	29	574 (399 to 826)	27	7.4% (4.9 to 11.1)		13	2.28 (1.52 to 3.27)
Unknown	127	2	587	1	170 (24 to 1208)	1	5.6% (0.8 to 33.4)		1	0.93 (0.02 to 5.19)
Proximal polyps††								0.03		
No	2475	40	11 207	25	223 (151 to 330)	23	2.4% (1.5 to 3.7)		25	1.00 (0.64 to 1.47)
Yes	3764	60	14 590	53	363 (278 to 476)	47	4.1% (2.9 to 5.7)		35	1.52 (1.14 to 1.99)
**After first surveillance (with one surveillance visit, censored at any second surveillance visit**)										
Total	3963	100	17 531	52	297 (226 to 389)	46	4.0% (2.8 to 5.8)		43	1.22 (0.91 to 1.60)
Sex								0.82		
Women	1367	34	6377	19	298 (190 to 467)	18	4.8% (2.7 to 8.6)		11	1.67 (1.00 to 2.61)
Men	2596	66	11 154	33	296 (210 to 416)	28	3.4% (2.1 to 5.5)		31	1.05 (0.73 to 1.48)
Age at baseline, years								0.08		
<55	616	16	2846	8	281 (141 to 562)	7	2.5% (1.1 to 5.9)		2	4.26 (1.84 to 8.39)
55–64	1299	33	5609	9	160 (83 to 308)	9	3.0% (1.2 to 7.7)		11	0.85 (0.39 to 1.61)
65–74	1529	39	6684	23	344 (229 to 518)	19	3.6% (2.1 to 6.1)		21	1.10 (0.70 to 1.65)
≥75	519	13	2392	12	502 (285 to 883)	11	7.9% (4.0 to 15.3)		9	1.29 (0.67 to 2.25)
No of PMPs								0.89		
1	295	7	1308	3	229 (74 to 711)	3	4.4% (1.2 to 15.0)		3	0.90 (0.19 to 2.63)
2	1521	38	7130	22	309 (203 to 469)	19	4.3% (2.4 to 7.6)		17	1.29 (0.81 to 1.95)
3	767	19	3314	8	241 (121 to 483)	8	3.3% (1.4 to 7.6)		8	1.00 (0.43 to 1.97)
4	402	10	1806	6	332 (149 to 739)	4	1.6% (0.5 to 4.8)		5	1.33 (0.49 to 2.89)
≥5	978	25	3973	13	327 (190 to 564)	12	5.5% (2.5 to 11.9)		10	1.33 (0.71 to 2.28)
PMP size, mm§								0.86		
<10	375	9	1637	6	367 (165 to 816)	6	5.6% (2.0 to 15.2)		4	1.54 (0.57 to 3.36)
10–19	1917	48	8757	24	274 (184 to 409)	22	3.4% (2.0 to 5.8)		21	1.15 (0.74 to 1.71)
≥20	1649	42	7068	22	311 (205 to 473)	18	4.3% (2.4 to 7.7)		18	1.24 (0.78 to 1.88)
Adenoma histology¶								0.22		
Tubular	1510	38	6820	13	191 (111 to 328)	12	2.2% (1.0 to 4.4)		16	0.81 (0.43 to 1.39)
Tubulovillous	1893	48	8293	29	350 (243 to 503)	26	5.9% (3.6 to 9.5)		20	1.42 (0.95 to 2.04)
Villous	443	11	1896	8	422 (211 to 844)	7	3.6% (1.7 to 7.7)		5	1.56 (0.67 to 3.07)
Unknown	117	3	522	2	383 (96 to 1533)	1	1.1% (0.2 to 7.8)		1	1.77 (0.21 to 6.38)
Adenoma dysplasia**								0.12		
Low grade	2945	74	13 079	32	245 (173 to 346)	28	3.7% (2.3 to 5.9)		31	1.03 (0.70 to 1.45)
High grade	927	23	3971	17	428 (266 to 689)	16	5.3% (2.7 to 10.3)		10	1.63 (0.95 to 2.61)
Unknown	91	2	481	3	623 (201 to 1933)	2	3.8% (0.9 to 15.3)		1	2.75 (0.57 to 8.04)
Proximal polyps††								0.10		
No	1546	39	7157	16	224 (137 to 365)	15	3.0% (1.5 to 5.7)		17	0.96 (0.55 to 1.55)
Yes	2417	61	10 374	36	347 (250 to 481)	31	4.7% (3.0 to 7.4)		26	1.39 (0.97 to 1.92)
**After second surveillance (with two or more surveillance visits, censored at end of follow-up**)										
Total	2259	100	14 990	32	213 (151 to 302)	25	2.3% (1.5 to 3.5)		39	0.82 (0.56 to 1.16)
Sex								0.57		
Women	741	33	5067	10	197 (106 to 367)	8	2.1% (1.0 to 4.3)		9	1.07 (0.51 to 1.97)
Men	1518	67	9923	22	222 (146 to 337)	17	2.4% (1.4 to 4.1)		30	0.74 (0.47 to 1.12)
Age at baseline, years								0.05		
<55	402	18	3036	3	99 (32 to 306)	3	1.6% (0.5 to 5.1)		3	0.96 (0.20 to 2.79)
55–64	834	37	5719	10	175 (94 to 325)	6	1.2% (0.5 to 3.0)		14	0.72 (0.35 to 1.32)
65–74	871	39	5450	16	294 (180 to 479)	13	3.5% (1.9 to 6.3)		19	0.86 (0.49 to 1.39)
≥75	152	7	785	3	382 (123 to 1185)	3	4.9% (1.2 to 18.4)		3	0.93 (0.19 to 2.73)
No of PMPs								0.31		
1	171	8	1266	5	395 (164 to 949)	4	3.2% (1.2 to 8.8)		3	1.51 (0.49 to 3.53)
2	793	35	5100	8	157 (78 to 314)	5	1.3% (0.5 to 3.4)		13	0.63 (0.27 to 1.24)
3	464	21	3092	7	226 (108 to 475)	5	1.9% (0.8 to 4.8)		8	0.87 (0.35 to 1.79)
4	242	11	1576	1	63 (9 to 450)	1	0.8% (0.1 to 5.2)		4	0.24 (0.01 to 1.35)
≥5	589	26	3957	11	278 (154 to 502)	10	4.1% (2.1 to 8.1)		11	1.02 (0.51 to 1.83)
PMP size, mm§								0.29		
<10	210	9	1450	3	207 (67 to 641)	3	2.6% (0.8 to 8.7)		4	0.81 (0.17 to 2.37)
10–19	1063	47	6698	9	134 (70 to 258)	7	1.1% (0.5 to 2.3)		17	0.52 (0.24 to 0.99)
≥20	968	43	6692	20	299 (193 to 463)	15	3.4% (1.9 to 5.8)		18	1.14 (0.70 to 1.76)
Adenoma histology¶								0.11		
Tubular	854	38	5704	8	140 (70 to 280)	7	1.2% (0.5 to 2.6)		15	0.55 (0.24 to 1.08)
Tubulovillous	1075	48	6999	14	200 (118 to 338)	10	1.9% (1.0 to 3.7)		18	0.77 (0.42 to 1.28)
Villous	259	11	1697	7	412 (197 to 865)	7	6.9% (3.1 to 15.1)		5	1.55 (0.62 to 3.19)
Unknown	71	3	589	3	509 (164 to 1578)	1	2.9% (0.4 to 19.1)		2	1.99 (0.41 to 5.83)
Adenoma dysplasia**								0.75		
Low grade	1681	74	11 004	22	200 (132 to 304)	18	2.0% (1.2 to 3.3)		28	0.78 (0.49 to 1.18)
High grade	525	23	3509	8	228 (114 to 456)	7	3.5% (1.6 to 7.8)		9	0.85 (0.37 to 1.67)
Unknown	53	2	477	2	419 (105 to 1675)	0	–		1	1.50 (0.18 to 5.41)
Proximal polyps††								0.21		
No	853	38	5758	9	156 (81 to 300)	6	1.2% (0.5 to 2.8)		15	0.62 (0.28 to 1.18)
Yes	1406	62	9232	23	249 (166 to 375)	19	2.9% (1.8 to 4.8)		24	0.94 (0.60 to 1.41)

High-risk patients were those with ≥2 PMPs, of which ≥1 was advanced, ≥5 PMPs or ≥1 large (≥20 mm) non-pedunculated PMP.

*Cumulative CRC incidence was estimated using the Kaplan-Meier method.

†P values were calculated with the log-rank test to compare cumulative CRC incidence among each category of the specified variable.

‡Numbers of expected CRCs were calculated by multiplying the 5-year age-group and sex-specific observed person-years by the corresponding CRC incidence rates in the general population of England in 2007.

§PMP size was defined according to the largest PMP seen at baseline. Patients with PMPs of unknown size are not included in the table; in the analyses without surveillance, there were 32 such patients with no CRC cases; in the analyses with one surveillance visit, there were 22 such patients with no CRC cases; and in the analyses with two or more surveillance visits, there were 18 such patients with no CRC cases.

¶Adenoma histology was defined according to the greatest degree of villousness seen at baseline.

**Adenoma dysplasia was defined according to the highest grade of dysplasia seen at baseline.

††Proximal polyps were defined as those proximal to the descending colon.

CRC, colorectal cancer; PMP, premalignant polyp; SIR, standardised incidence ratio.

Among low-risk patients, the median age was 64 years (IQR 55–72), 45% were female (table 4) and 51% attended ≥1 surveillance visits (table 3). The median time from baseline to first surveillance was 3.1 years (IQR 2.1–4.9). Over a median follow-up of 10.3 years (IQR 7.7–12.9), 206 CRCs were diagnosed, giving an incidence rate of 135 per 100 000 person-years (95% CI 118 to 155) (table 3).

Among high-risk patients, the median age was 67 years (IQR 60–73), 36% were female (table 5) and 64% attended ≥1 surveillance visits (table 3). The median time from baseline to first surveillance was 2.1 years (IQR 1.1–3.2). Over a median follow-up of 9.6 years (IQR 6.5–12.1), 162 CRCs were diagnosed, giving an incidence rate of 278 per 100 000 person-years (95% CI 238 to 324) (table 3). The two risk groups differed significantly on all baseline characteristics and high-risk patients had more surveillance than low-risk patients (online supplemental table 2).

In both risk groups, surveillance was associated with reduced CRC incidence. Among low-risk patients, CRC incidence was lower with ≥1 surveillance visits than with none, adjusting for characteristics associated with CRC incidence in the whole cohort (HR 0.58, 95% CI 0.41 to 0.83 for 1 visit; 0.53, 0.33 to 0.83 for ≥2 visits). A similar pattern was observed for high-risk patients (HR 0.71, 95% CI 0.49 to 1.03 for 1 visit; 0.44, 0.28 to 0.70 for ≥2 visits), although the CI of the HR for a single visit included one (table 3).

Among low-risk patients, without surveillance, cumulative CRC incidence at 10 years was 1.6% (95% CI 1.3% to 1.9%) (table 4; figure 2B) and CRC incidence was lower than in the general population (SIR 0.75, 95% CI 0.63 to 0.88). The CIs of all SIRs were below or crossed one, showing that CRC incidence was not elevated by any baseline characteristic (table 4).

Among high-risk patients, without surveillance, cumulative CRC incidence at ten years was 3.3% (95% CI 2.5% to 4.3%) (table 5; figure 2B) and CRC incidence was higher than in the general population (SIR 1.30, 95% CI 1.03 to 1.62) (table 5). Examining SIRs by baseline characteristics, CRC incidence without surveillance was higher than in the general population among women (SIR 1.79, 95% CI 1.23 to 2.51) and those with PMPs ≥20 mm (1.52, 1.06 to 2.11), adenomas with high-grade dysplasia (2.28, 1.52 to 3.27), or proximal polyps (1.52, 1.14 to 1.99) at baseline (table 5).

After a single surveillance visit, among high-risk patients, cumulative CRC incidence at 10 years was 4.0% (95% CI 2.8% to 5.8%) (table 5; figure 2D); higher than without surveillance, likely because the cohort had aged. Incidence of CRC was no longer significantly higher than in the general population (SIR 1.22, 95% CI 0.91 to 1.60). Examining SIRs by baseline characteristics, CRC incidence was higher than in the general population among women (SIR 1.67, 95% CI 1.00 to 2.61) and those aged <55 years (4.26, 1.84 to 8.39); however, these estimates were based on few CRC cases (table 5). After second surveillance, the CIs of all SIRs included one (table 5).

Results followed the same pattern when we did not exclude CRCs assumed to have arisen from incompletely excised baseline lesions. For some baseline polyp characteristics, there were slight changes to the associated p values in our analyses of CRC incidence or SIRs such that they became significant; for example, in the whole cohort, presence of ≥4 PMPs, PMPs ≥20 mm, adenomas with villous histology and proximal polyps became associated with elevated SIRs in the absence of surveillance, while in high-risk patients, this was seen for ≥4 PMPs and adenomas with tubulovillous/villous histology (online supplemental tables 3–7).

## Discussion

This study provides unique data on long-term post-polypectomy CRC incidence by baseline characteristics and a vitally important examination of the 2020 UK surveillance guidelines. Through investigation of 21 318 patients who underwent colonoscopy with polypectomy and were followed-up for a median of 10.1 years, we found that CRC incidence in most patients was similar to or lower than that in the general population. We demonstrated that the new UK guidelines are accurate at identifying and discriminating between those at increased risk of CRC who require surveillance, and those at low risk who can be managed by population-based non-invasive CRC screening instead.^6^

We identified several baseline risk factors for CRC, including older age (≥55 years) and presence of multiple (≥2) PMPs, adenomas with tubulovillous/villous/unknown histology or high-grade dysplasia, proximal polyps and a baseline visit spanning 2–90 days. This is in line with our previous studies which found associations between these factors and increased CRC incidence when this same cohort was stratified into risk groups following the 2002 UK guidelines,^9 10 18^ and other studies describing these as risk factors for metachronous advanced neoplasia.^6^ However, compared with the general population, CRC incidence was higher only among those with adenomas with high-grade dysplasia or ≥2 PMPs, of which ≥1 was advanced at baseline (29% of our cohort). This is important because in a resource-constrained setting, and given the serious, although rare, complications of colonoscopy due to its invasive nature,^23 24^ surveillance should be directed towards patients at higher CRC risk than the general population after polypectomy.^6^

Applying the risk classification criteria in the 2020 UK guidelines,^6^ 29% of patients were classified as high risk, the same proportion as that identified as being at increased risk in our analyses of SIRs by baseline characteristics. Among these patients, CRC incidence without surveillance was 1.3 times higher than in the general population. Incidence was elevated to a larger extent in women than men, although the CIs of the SIRs overlapped. The elevated risk among these high-risk patients appeared to be largely driven by the presence of PMPs ≥20 mm, adenomas with high-grade dysplasia, and proximal polyps, which warrant close attention from endoscopists. The excess risk was eliminated after first surveillance, indicating that the guideline recommendation for a one-off surveillance colonoscopy is appropriate.

The increased CRC risk associated with PMPs ≥20 mm, adenomas with high-grade dysplasia, and proximal polyps might partly be the result of incomplete excision because the risk of incomplete excision is greater for advanced, large or proximal polyps.^25 26^ Unfortunately, histological completeness of excision was not consistently recorded in our data and so we were unable to explore this hypothesis.

Among low-risk patients, CRC incidence without surveillance was lower than in the general population. Therefore, it is appropriate that this group are recommended to participate in their national CRC screening programme when invited rather than undergo surveillance, thereby minimising exposure of low-risk patients to unnecessary invasive surveillance procedures and alleviating pressures on endoscopy services. In the UK, screening involves the stool-based faecal immunochemical test, currently offered biennially to people aged 60–74 years (50–74 years in Scotland).^27 28^ In this way, the new guidelines are expected to reduce surveillance colonoscopy workload by up to 80%, compared with practice under the 2002 UK guidelines,^2^ although they will still ensure that high-risk patients are captured and receive surveillance.^6^

The 2020 UK guidelines are an improvement on the 2002 guidelines because they incorporate additional data on long-term post-polypectomy CRC outcomes.^2 6^ This is also true for the EU and US surveillance guidelines which were updated in 2020.^7 8^ However, there is still a lack of high-quality studies with CRC incidence or mortality as endpoints. Apart from the present study and our two previous analyses using this cohort,^9 10 18^ only one other has compared post-polypectomy CRC incidence with that in the general population, in the absence and presence of surveillance.^29^ Cottet *et al* reported that, compared with the general population, CRC incidence was four times higher among patients with baseline adenomas ≥10 mm, with villous features, or high-grade dysplasia without surveillance, but similar with ≥1 surveillance visits. In contrast, CRC incidence among patients with tubular adenomas <10 mm was comparable to that in the general population regardless of exposure to surveillance. However, this study had a small sample size (n=5779) and baseline colonoscopies were performed from 1990 to 1999, predating colonoscopy quality improvements.^29^

A further three studies examining post-polypectomy CRC incidence were published in 2020.^15–17^ The findings from two of these indicate that, compared with patients with normal colonoscopy findings (‘no adenomas’ or ‘no polyps’), patients with baseline adenomas ≥10 mm, with villous features, or high-grade dysplasia, or serrated polyps ≥10 mm are at increased CRC risk, whereas patients with tubular adenomas or serrated polyps <10 mm are not.^15 17^ In the third study, compared with the general population, CRC incidence was two times higher among patients with baseline adenomas ≥20 mm; similar among those with adenomas with high-grade dysplasia; and two-thirds lower among those with adenomas <20 mm with low-grade dysplasia.^16^ These studies did not estimate CRC incidence without surveillance, which is a major limitation because surveillance differed in intensity and likely differentially affected CRC outcomes between the compared groups.

Serrated polyps have increasingly been recognised as important CRC precursors over the last two decades,^30^ but their natural history remains unclear because they have been examined in few long-term studies. Until recently, there was a lack of consensus regarding the nomenclature and histological classification of serrated polyps.^30^ Therefore, these lesions were likely under-recorded and misclassified in our dataset and so our serrated polyp data should be interpreted with caution. Moreover, all patients included as having serrated polyps in our dataset also had an adenoma at baseline, which might not be representative of a real-life population of patients with serrated polyps.

The observational design of our study means we cannot infer causality from the associations between baseline characteristics and CRC incidence. Moreover, this design is not necessarily ideally suited for determining optimal surveillance intervals. Randomised controlled trials comparing different surveillance intervals with CRC incidence as the endpoint, such as the FORTE (Five OR TEn year colonoscopy for 1–2 non-advanced adenomas) and EPoS (European Polyp Surveillance) trials,^31 32^ will provide additional data to inform whether the surveillance intervals recommended in the 2020 UK, EU and US guidelines are appropriate.

Another limitation is that as most examinations in our data occurred during the era of the 2002 UK guidelines,^2^ surveillance regimens advised for our cohort differed from current recommendations. Adherence to the guidelines was not complete,^18^ and the amount and frequency of surveillance varied across patients. To mitigate the effects of any associated bias, we controlled for number of surveillance visits in our analyses. We had incomplete information on why patients were attending follow-up examinations; therefore, some ‘surveillance’ examinations might have been for symptomatic purposes. Furthermore, we had no information on reasons for non-attendance at surveillance. It is possible that some patients underwent surveillance at hospitals other than those from which we obtained data. Baseline data were more frequently missing for patients attending surveillance compared with non-attenders which might have introduced bias. Our use of routinely collected data means that misclassification is likely present in the dataset. Finally, we might be overestimating CRC incidence in the general population as compared with our cohort; while we excluded patients who had CRC at or before baseline colonoscopy from our cohort, this exclusion did not apply to the general population.

Strengths include the large size, nationwide design and detailed information on baseline patient, procedural, and polyp characteristics and surveillance examinations. There were few missing data and losses to follow-up were minimal. We restricted our dataset to patients with a high-quality baseline colonoscopy and so the findings are applicable to contemporary colonoscopy practice. We used the definitive endpoint of CRC incidence and accounted for the effects of surveillance on our incidence estimates; this enabled us to elucidate the effects of individual baseline characteristics on long-term post-polypectomy CRC incidence.

## Conclusion

Our findings demonstrate that the 2020 UK guidelines accurately identify patients at high risk of CRC after polypectomy, and that the recommendation for a one-off surveillance colonoscopy seems appropriate for these patients and would help eliminate their excess risk. Moreover, these guidelines will ensure that low-risk patients, who we showed are very unlikely to develop CRC after polypectomy, are not exposed to unnecessary surveillance colonoscopies and are appropriately managed by population-based non-invasive CRC screening instead.

## Data Availability

Data are available upon reasonable request. We may be able to share de-identified participant data with researchers following publication of this manuscript. Requests for data should be directed to the corresponding author. Data sharing will need to be approved by third party data providers.
