# Supplementary material for: Colorectal cancer risk following polypectomy in a multicentre, retrospective, cohort study: an evaluation of the 2020 UK post-polypectomy surveillance guidelines
Source: Gut. 2021 Mar 5;70(12):2307–20. doi: 10.1136/gutjnl-2020-323411 (PMC8588296; doi:10.1136/gutjnl-2020-323411)
Supplement: Supplementary data [file gutjnl-2020-323411supp001.pdf]

Supplementary Table 1. Baseline characteristics by attendance at surveillance (n=21,318)

	Attended ≥1 surveillance visit		Attended no surveillance visits		P value[a]
	n	(%)	n	(%)	
Total	11,604	(54.4)	9,714	(45.6)	
Sex					0.003
Women	4,804	(41.4)	4,218	(43.4)	
Men	6,800	(58.6)	5,496	(56.6)	
Age at baseline, years					<0.001
<55	2,702	(23.3)	1,596	(16.4)	
55-64	3,799	(32.7)	2,157	(22.2)	
65-74	3,780	(32.6)	3,114	(32.1)	
≥75	1,323	(11.4)	2,847	(29.3)	
No. of PMPs					<0.001
1	6,188	(53.3)	6,043	(62.2)	
2-4	4,438	(38.2)	3,262	(33.6)	
≥5	978	(8.4)	409	(4.2)	
PMP size, mm[b]					<0.001
<10	5,608	(48.3)	5,945	(61.2)	
10-19	3,591	(30.9)	2,490	(25.6)	
≥20	2,366	(20.4)	1,259	(13.0)	
Unknown	39	(0.3)	20	(0.2)	
Adenoma histology[c]					<0.001
Tubular	6,526	(56.2)	6,260	(64.4)	
Tubulovillous	3,849	(33.2)	2,631	(27.1)	
Villous	660	(5.7)	385	(4.0)	
Unknown	569	(4.9)	438	(4.5)	
Adenoma dysplasia[d]					<0.001
Low-grade	9,857	(84.9)	8,735	(89.9)	
High-grade	1,389	(12.0)	759	(7.8)	
Unknown	358	(3.1)	220	(2.3)	
Proximal polyps[e]					0.005
No	6,195	(53.4)	5,371	(55.3)	
Yes	5,409	(46.6)	4,343	(44.7)	
Year of baseline visit					<0.001
1984-1999	1,384	(11.9)	673	(6.9)	
2000-2004	3,793	(32.7)	2,858	(29.4)	
2005-2010	6,427	(55.4)	6,183	(63.7)	
Length of baseline visit, days					<0.001

1	7,369	(63.5)	6,854	(70.6)	
2-90	1,732	(14.9)	1,303	(13.4)	
91-183	1,269	(10.9)	816	(8.4)	
≥184	1,234	(10.6)	741	(7.6)	
Family history of cancer/CRC[f]					<0.001
No	10,430	(89.9)	9,300	(95.7)	
Yes	1,174	(10.1)	414	(4.3)	

PMP=premalignant polyp. CRC=colorectal cancer.
[a]P values were calculated with the chi-square test to compare patients with and without surveillance.
[b]PMP size was defined according to the largest PMP seen at baseline.
[c]Adenoma histology was defined according to the greatest degree of villousness seen at baseline.
[d]Adenoma dysplasia was defined according to the highest grade of dysplasia seen at baseline.
[e]Proximal polyps were defined as those proximal to the descending colon.
[f]Family history of cancer/CRC was defined as 'family history of cancer or CRC reported at an examination before or during visit'. Of cases with a 'family history of cancer', 72% were from a specialist hospital for colorectal diseases and so we assumed these cases had a family history of CRC.

Supplementary Table 2. Number of surveillance visits and baseline characteristics by risk group (n=21,318)

	Low-risk patients		High-risk patients		
	n	(%)	N	(%)	P value[a]
Total	15,079	(70.7)	6,239	(29.3)	
No. of surveillance visits					<0.001
0	7,438	(49.3)	2,276	(36.5)	
1	4,199	(27.8)	1,704	(27.3)	
2	2,254	(14.9)	1,261	(20.2)	
≥3	1,188	(7.9)	998	(16.0)	
Sex					<0.001
Women	6,796	(45.1)	2,226	(35.7)	
Men	8,283	(54.9)	4,013	(64.3)	
Age at baseline, years					<0.001
<55	3,469	(23.0)	829	(13.3)	
55-64	4,193	(27.8)	1,763	(28.3)	
65-74	4,589	(30.4)	2,305	(36.9)	
≥75	2,828	(18.8)	1,342	(21.5)	
No. of PMPs					<0.001
1	11,733	(77.8)	498	(8.0)	
2-4	3,346	(22.2)	4,354	(69.8)	
≥5	0	(0.0)	1,387	(22.2)	
PMP size, mm[b]					<0.001
<10	10,985	(72.8)	568	(9.1)	
10-19	2,981	(19.8)	3,100	(49.7)	
≥20	1,086	(7.2)	2,539	(40.7)	
Unknown	27	(0.2)	32	(0.5)	
Adenoma histology[c]					<0.001
Tubular	10,376	(68.8)	2,410	(38.6)	
Tubulovillous	3,517	(23.3)	2,963	(47.5)	
Villous	359	(2.4)	686	(11.0)	
Unknown	827	(5.5)	180	(2.9)	
Adenoma dysplasia[d]					<0.001
Low-grade	13,888	(92.1)	4,704	(75.4)	
High-grade	740	(4.9)	1,408	(22.6)	
Unknown	451	(3.0)	127	(2.0)	
Proximal polyps[e]					<0.001
No	9,091	(60.3)	2,475	(39.7)	
Yes	5,988	(39.7)	3,764	(60.3)	
Year of baseline visit					0.002

1984-1999	1,404	(9.3)	653	(10.5)	
2000-2004	4,792	(31.8)	1,859	(29.8)	
2005-2010	8,883	(58.9)	3,727	(59.7)	
Length of baseline visit, days					<0.001
1	11,336	(75.2)	2,887	(46.3)	
2-90	1,750	(11.6)	1,285	(20.6)	
91-183	1,108	(7.3)	977	(15.7)	
≥184	885	(5.9)	1,090	(17.5)	
Family history of cancer/CRC[f]					<0.001
No	13,757	(91.2)	5,973	(95.7)	
Yes	1,322	(8.8)	266	(4.3)	
Follow-up time, years (median [IQR])	10.3 (7.7-12.9)		9.6 (6.5-12.1)		

PMP=premalignant polyp. CRC=colorectal cancer.
[a]P values were calculated with the chi-square test to compare low-risk and high-risk patients.
[b]PMP size was defined according to the largest PMP seen at baseline.
[c]Adenoma histology was defined according to the greatest degree of villousness seen at baseline.
[d]Adenoma dysplasia was defined according to the highest grade of dysplasia seen at baseline.
[e]Proximal polyps were defined as those proximal to the descending colon.
[f]Family history of cancer/CRC was defined as ‘family history of cancer or CRC reported at an examination before or during visit’. Of cases with a ‘family history of cancer’, 72% were from a specialist hospital for colorectal diseases and so we assumed these cases had a family history of CRC.

Supplementary Table 3. Long-term incidence of colorectal cancer by number of surveillance visits and baseline characteristics, without excluding colorectal cancers assumed to have arisen from incompletely excised lesions (n=21,318)

	n (%)	No. of person-years	No. of CRCs	Incidence rate per 100,000 person-years (95% CI)	Univariable HR (95% CI)	P value[a]	Multivariable HR (95% CI)[b]	P value[a]
Total	21,318 (100)	210,814	393	186 (169-206)				
No. of surveillance visits[c]						<0.001		<0.001
0	9,714 (45.6)	116,248	233	200 (176-228)	1		1	
1	5,903 (27.7)	56,923	101	177 (146-216)	0.73 (0.57-0.93)		0.64 (0.50-0.82)	
2	3,515 (16.5)	25,058	33	132 (94-185)	0.50 (0.34-0.73)		0.42 (0.29-0.62)	
≥3	2,186 (10.3)	12,586	26	207 (141-303)	0.67 (0.43-1.04)		0.52 (0.33-0.82)	
Sex						0.64		0.59
Women	9,022 (42.3)	92,173	168	182 (157-212)	1		1	
Men	12,296 (57.7)	118,641	225	190 (166-216)	1.05 (0.86-1.28)		1.06 (0.86-1.30)	
Age at baseline, years						<0.001		<0.001
<55	4,298 (20.2)	51,463	37	72 (52-99)	1		1	
55-64	5,956 (27.9)	64,938	82	126 (102-157)	1.81 (1.23-2.67)		1.65 (1.12-2.43)	
65-74	6,894 (32.3)	65,186	165	253 (217-295)	3.79 (2.65-5.43)		3.22 (2.25-4.63)	
≥75	4,170 (19.6)	29,228	109	373 (309-450)	6.00 (4.11-8.75)		4.45 (3.03-6.52)	
No. of PMPs						<0.001		0.004
1	12,231 (57.4)	124,117	170	137 (118-159)	1		1	
2	4,714 (22.1)	45,601	106	232 (192-281)	1.72 (1.35-2.19)			
3	2,035 (9.6)	19,482	46	236 (177-315)	1.75 (1.26-2.43)		}1.35 (1.08-1.70)	
4	951 (4.5)	8,856	26	294 (200-431)	2.18 (1.44-3.30)			
≥5	1,387 (6.5)	12,760	45	353 (263-472)	2.64 (1.90-3.67)		1.76 (1.23-2.53)	
PMP size, mm[d]						<0.001		0.03
<10	11,553 (54.2)	116,281	166	143 (123-166)	1		1	
10-19	6,081 (28.5)	59,382	110	185 (154-223)	1.30 (1.02-1.65)		1.04 (0.80-1.36)	
≥20	3,625 (17.0)	34,544	116	336 (280-403)	2.36 (1.86-2.99)		1.52 (1.12-2.05)	
Unknown	59 (0.3)	607	1	165 (23-1,169)	1.12 (0.16-7.98)		0.71 (0.10-5.15)	
Adenoma histology[e]						<0.001		<0.001
Tubular	12,786 (60.0)	127,882	173	135 (117-157)	1		1	
Tubulovillous	6,480 (30.4)	62,187	153	246 (210-288)	1.83 (1.47-2.28)		1.51 (1.20-1.89)	
Villous	1,045 (4.9)	9,958	38	382 (278-524)	2.84 (2.00-4.04)		1.80 (1.24-2.62)	
Unknown	1,007 (4.7)	10,787	29	269 (187-387)	1.93 (1.30-2.86)		2.06 (1.37-3.11)	
Adenoma dysplasia[f]						<0.001		0.003
Low-grade	18,592 (87.2)	183,696	302	164 (147-184)	1		1	
High-grade	2,148 (10.1)	19,913	76	382 (305-478)	2.34 (1.82-3.01)		1.63 (1.24-2.14)	

Unknown	578 (2.7)	7,206	15	208 (125-345)	1.19 (0.71-2.00)		1.22 (0.71-2.12)	
Proximal polyps[g]						<0.001		<0.001
No	11,566 (54.3)	118,513	161	136 (116-159)	1		1	
Yes	9,752 (45.8)	92,301	232	251 (221-286)	1.88 (1.54-2.30)		1.67 (1.34-2.07)	
Year of baseline visit						0.84		0.40
1984-1999	2,057 (9.7)	28,319	63	222 (174-285)	1		1	
2000-2004	6,651 (31.2)	74,494	143	192 (163-226)	0.94 (0.69-1.30)		0.87 (0.63-1.21)	
2005-2010	12,610 (59.2)	108,001	187	173 (150-200)	0.91 (0.66-1.26)		0.80 (0.57-1.11)	
Length of baseline visit, days						<0.001		0.003
1	14,223 (66.7)	140,884	209	148 (130-170)	1		1	
2-90	3,035 (14.2)	29,429	78	265 (212-331)	1.80 (1.39-2.33)		1.62 (1.23-2.12)	
91-183	2,085 (9.8)	21,071	49	233 (176-308)	1.57 (1.15-2.14)		1.33 (0.96-1.84)	
≥184	1,975 (9.3)	19,430	57	293 (226-380)	1.97 (1.47-2.64)		1.49 (1.09-2.04)	
Family history of cancer/CRC[h]						0.12		0.11
No	19,730 (92.6)	191,764	365	190 (172-211)	1		1	
Yes	1,588 (7.5)	19,051	28	147 (101-213)	0.75 (0.51-1.10)		1.40 (0.94-2.09)	

CRC=colorectal cancer. CI=confidence interval. HR=hazard ratio. PMP=premalignant polyp. mm=millimetre.
[a]P values were calculated with the likelihood ratio test.
[b]The multivariable model contained the variables selected in the multivariable model in the main analysis (Table 1), i.e. number of surveillance visits, age, number of PMPs, adenoma histology, adenoma dysplasia, proximal polyps, and length of baseline visit. For these variables, the multivariable HRs were from the final multivariable model and the p values were for inclusion of the variable in the model. For the remaining variables, the multivariable HRs were for if the variable was added as an additional variable to the final multivariable model.
[c]Number of surveillance visits was included as a time-varying covariate, meaning that patients who had surveillance contributed person-years to more than a single category of number of surveillance visits.
[d]PMP size was defined according to the largest PMP seen at baseline.
[e]Adenoma histology was defined according to the greatest degree of villousness seen at baseline.
[f]Adenoma dysplasia was defined according to the highest grade of dysplasia seen at baseline.
[g]Proximal polyps were defined as those proximal to the descending colon.
[h]Family history of cancer/CRC was defined as 'family history of cancer or CRC reported at an examination before or during visit'. Of cases with a 'family history of cancer', 72% were from a specialist hospital for colorectal diseases and so we assumed these cases had a family history of CRC.

Supplementary Table 4. Cumulative incidence of colorectal cancer and age-sex-standardised incidence ratios in the whole cohort, without excluding colorectal cancers assumed to have arisen from incompletely excised lesions (n=21,318)

							At 10 years			Standardisation		
	n	(%)	No. of person-years	No. of CRCs	Incidence rate per 100,000 person-years (95% CI)		No. of CRCs	Cumulative incidence (95% CI)[a]	P value[b]	No. of expected CRCs[c]	SIR (95% CI)	
**After baseline (without surveillance, censored at any first surveillance visit)**												
Total	21,318	(100)	116,248	233	200	(176-228)	202	2.1% (1.8-2.4)		242	0.96	(0.84-1.09)
Sex									0.42			
Women	9,022	(42)	52,431	100	191	(157-232)	81	1.8% (1.4-2.3)		87	1.16	(0.94-1.41)
Men	12,296	(58)	63,816	133	208	(176-247)	121	2.3% (1.9-2.8)		156	0.85	(0.72-1.01)
Age at baseline, years									<0.001			
<55	4,298	(20)	26,718	12	45	(26-79)	9	0.4% (0.2-0.8)		13	0.93	(0.48-1.63)
55-64	5,956	(28)	32,358	41	127	(93-172)	35	1.5% (1.0-2.1)		51	0.81	(0.58-1.10)
65-74	6,894	(32)	35,831	98	274	(224-333)	85	2.7% (2.1-3.4)		100	0.98	(0.80-1.19)
≥75	4,170	(20)	21,341	82	384	(309-477)	73	3.9% (3.0-5.1)		79	1.04	(0.83-1.30)
No. of PMPs									<0.001			
1	12,231	(57)	72,860	108	148	(123-179)	88	1.5% (1.2-1.8)		144	0.75	(0.61-0.90)
2	4,714	(22)	24,974	62	248	(194-318)	54	2.5% (1.8-3.3)		56	1.11	(0.85-1.43)
3	2,035	(10)	9,612	25	260	(176-385)	23	3.2% (2.0-4.9)		22	1.13	(0.73-1.67)
4	951	(4)	3,971	17	428	(266-689)	17	6.0% (3.5-10.2)		9	1.88	(1.09-3.00)
≥5	1,387	(7)	4,830	21	435	(283-667)	20	4.0% (2.4-6.9)		11	1.92	(1.19-2.94)
PMP size, mm[d]									<0.001			
<10	11,553	(54)	72,061	112	155	(129-187)	95	1.6% (1.3-2.0)		145	0.77	(0.64-0.93)
10-19	6,081	(29)	29,408	62	211	(164-270)	52	2.2% (1.6-3.1)		64	0.97	(0.75-1.25)
≥20	3,625	(17)	14,553	58	399	(308-516)	54	3.8% (2.8-5.2)		33	1.75	(1.33-2.27)
Adenoma histology[e]									<0.001			
Tubular	12,786	(60)	75,483	119	158	(132-189)	102	1.6% (1.3-2.0)		153	0.78	(0.64-0.93)
Tubulovillous	6,480	(30)	30,698	79	257	(206-321)	69	2.6% (2.0-3.4)		68	1.16	(0.92-1.45)
Villous	1,045	(5)	4,505	20	444	(286-688)	19	4.2% (2.5-7.0)		11	1.84	(1.12-2.84)
Unknown	1,007	(5)	5,562	15	270	(163-447)	12	3.1% (1.7-5.8)		10	1.45	(0.81-2.40)
Adenoma dysplasia[f]									<0.001			
Low-grade	18,592	(87)	104,400	182	174	(151-202)	154	1.7% (1.5-2.1)		215	0.85	(0.73-0.98)
High-grade	2,148	(10)	8,373	45	537	(401-720)	43	6.0% (4.3-8.4)		20	2.24	(1.63-2.99)
Unknown	578	(3)	3,475	6	173	(78-384)	5	2.2% (0.8-5.8)		7	0.87	(0.32-1.89)
Proximal polyps[g]									<0.001			
No	11,566	(54)	67,073	94	140	(114-172)	83	1.6% (1.2-2.0)		133	0.71	(0.57-0.87)

Yes	9,752	(46)	49,174	139	283	(239-334)	119	2.7% (2.2-3.3)		110	1.27	(1.07-1.50)
No. of APMPs and PMPs									<0.001			
No APMPs, 1 PMP	7,506	(35)	49,423	66	134	(105-170)	53	1.3% (1.0-1.8)		96	0.69	(0.53-0.88)
No APMPs, 2-4 PMPs	3,346	(16)	19,581	38	194	(141-267)	34	2.2% (1.6-3.2)		43	0.89	(0.63-1.22)
No APMPs, ≥5 PMPs	461	(2)	1,991	3	151	(49-467)	3	1.4% (0.4-4.5)		4	0.73	(0.15-2.14)
1 APMP, no other PMPs	4,725	(22)	23,437	42	179	(132-242)	35	1.8% (1.2-2.6)		49	0.86	(0.62-1.16)
≥1 APMP, ≥2 total PMPs	5,280	(25)	21,815	84	385	(311-477)	77	4.0% (3.1-5.2)		51	1.65	(1.31-2.04)
**After first surveillance (with one or more surveillance visits, censored at end of follow-up)**												
Total	11,604	(100)	94,567	160	169	(145-198)	128	1.7% (1.4-2.0)		213	0.75	(0.64-0.88)
Sex									0.97			
Women	4,804	(41)	39,742	68	171	(135-217)	56	1.9% (1.4-2.5)		67	1.02	(0.79-1.29)
Men	6,800	(59)	54,825	92	168	(137-206)	72	1.6% (1.2-2.0)		146	0.63	(0.51-0.77)
Age at baseline, years									<0.001			
<55	2,702	(23)	24,746	25	101	(68-150)	20	0.9% (0.6-1.4)		19	1.30	(0.84-1.93)
55-64	3,799	(33)	32,580	41	126	(93-171)	30	1.2% (0.8-1.8)		69	0.60	(0.43-0.81)
65-74	3,780	(33)	29,354	67	228	(180-290)	54	2.4% (1.8-3.2)		95	0.71	(0.55-0.90)
≥75	1,323	(11)	7,887	27	342	(235-499)	24	3.9% (2.5-6.2)		30	0.89	(0.59-1.30)
No. of PMPs									<0.001			
1	6,188	(53)	51,257	62	121	(94-155)	52	1.3% (1.0-1.7)		108	0.58	(0.44-0.74)
2	2,617	(23)	20,626	44	213	(159-287)	31	1.8% (1.2-2.6)		48	0.91	(0.66-1.23)
3	1,225	(11)	9,870	21	213	(139-326)	17	1.9% (1.2-3.1)		24	0.87	(0.54-1.33)
4	596	(5)	4,884	9	184	(96-354)	6	1.2% (0.5-2.7)		12	0.73	(0.33-1.38)
≥5	978	(8)	7,930	24	303	(203-452)	22	4.0% (2.5-6.3)		21	1.17	(0.75-1.74)
PMP size, mm[d]									<0.001			
<10	5,608	(48)	44,221	54	122	(94-159)	44	1.3% (0.9-1.7)		93	0.58	(0.43-0.75)
10-19	3,591	(31)	29,974	48	160	(121-212)	40	1.5% (1.1-2.1)		70	0.69	(0.51-0.91)
≥20	2,366	(20)	19,991	58	290	(224-375)	44	2.9% (2.1-4.0)		48	1.20	(0.91-1.55)
Adenoma histology[e]									<0.001			
Tubular	6,526	(56)	52,399	54	103	(79-135)	42	0.9% (0.7-1.3)		114	0.48	(0.36-0.62)
Tubulovillous	3,849	(33)	31,489	74	235	(187-295)	62	2.5% (2.0-3.3)		74	1.01	(0.79-1.26)
Villous	660	(6)	5,453	18	330	(208-524)	14	3.2% (1.8-5.6)		14	1.28	(0.76-2.02)
Unknown	569	(5)	5,225	14	268	(159-452)	10	2.6% (1.4-5.0)		11	1.23	(0.67-2.06)
Adenoma dysplasia[f]									0.01			
Low-grade	9,857	(85)	79,296	120	151	(127-181)	95	1.5% (1.2-1.9)		175	0.69	(0.57-0.82)
High-grade	1,389	(12)	11,539	31	269	(189-382)	28	2.9% (2.0-4.3)		29	1.06	(0.72-1.50)
Unknown	358	(3)	3,731	9	241	(126-464)	5	1.8% (0.7-4.3)		8	1.10	(0.50-2.09)
Proximal polyps[g]									<0.001			
No	6,195	(53)	51,440	67	130	(103-165)	53	1.3% (1.0-1.7)		109	0.61	(0.48-0.78)
Yes	5,409	(47)	43,126	93	216	(176-264)	75	2.2% (1.7-2.8)		103	0.90	(0.73-1.10)
No. of APMPs and PMPs									<0.001			

No APMPs, 1 PMP	3,402	(29)	26,997	27	100	(69-146)	23	1.1% (0.7-1.7)	54	0.50	(0.33-0.73)
No APMPs, 2-4 PMPs	1,748	(15)	13,362	17	127	(79-205)	11	1.0% (0.5-1.9)	30	0.57	(0.33-0.91)
No APMPs, ≥5 PMPs	310	(3)	2,566	6	234	(105-520)	6	3.1% (1.4-7.2)	6	0.95	(0.35-2.06)
1 APMP, no other PMPs	2,786	(24)	24,259	35	144	(104-201)	29	1.5% (1.1-2.2)	54	0.65	(0.46-0.91)
≥1 APMP, ≥2 total PMPs	3,358	(29)	27,382	75	274	(218-343)	59	2.6% (2.0-3.4)	69	1.09	(0.86-1.37)

CRC=colorectal cancer. CI=confidence interval. SIR=standardised incidence ratio. PMP=premalignant polyp. APMP=advanced PMP.
[a]Cumulative CRC incidence was estimated using the Kaplan-Meier method.
[b]P values were calculated with the log-rank test to compare cumulative CRC incidence among each category of the specified variable.
[c]Numbers of expected CRCs were calculated by multiplying the 5-year age-group and sex-specific observed person-years by the corresponding CRC incidence rates in the general population of England in 2007.
[d]PMP size was defined according to the largest PMP seen at baseline. Patients with PMPs of unknown size are not included in the table; in the analyses without surveillance, there were 59 such patients, of whom one was diagnosed with CRC; and in the analyses with one or more surveillance visits, there were 39 such patients with no CRC cases.
[e]Adenoma histology was defined according to the greatest degree of villousness seen at baseline.
[f]Adenoma dysplasia was defined according to the highest grade of dysplasia seen at baseline.
[g]Proximal polyps were defined as those proximal to the descending colon.

Supplementary Table 5. Effect of surveillance on colorectal cancer incidence by number of surveillance visits and risk group, without excluding colorectal cancers assumed to have arisen from incompletely excised lesions (n=21,318)

	n	(%)	No. of person-years	No. of CRCs	Incidence rate per 100,000 person-years (95% CI)		Effect of surveillance on CRC incidence[a]			
							Univariable HR (95% CI)	P value[b]	Multivariable HR (95% CI)[c]	P value[b]
Low-risk patients[d]	..	..	..	..	..	..	..	<0.001		0.001
0 visits	7,438	(49.3)	90,451	138	153	(129-180)	1	..	1	
1 visit	4,199	(27.8)	39,392	44	112	(83-150)	0.56 (0.40-0.80)	..	0.58 (0.41-0.82)	
≥2 visits	3,442	(22.8)	22,654	26	115	(78-169)	0.48 (0.30-0.75)	..	0.52 (0.33-0.83)	
Total	15,079	(70.7)	152,497	208	136	(119-156)	..	..		
High-risk patients[d]	..	..	..	..	..	..	..	<0.001	..	<0.001
0 visits	2,276	(36.5)	25,796	95	368	(301-450)	1	..	1	..
1 visit	1,704	(27.3)	17,531	57	325	(251-422)	0.71 (0.51-1.01)	..	0.69 (0.49-0.98)	..
≥2 visits	2,259	(36.2)	14,990	33	220	(157-310)	0.42 (0.27-0.65)	..	0.43 (0.28-0.68)	..
Total	6,239	(29.3)	58,318	185	317	(275-366)	..	..		

CRC=colorectal cancer. CI=confidence interval. HR=hazard ratio.

[a]Number of surveillance visits was included as a time-varying covariate, meaning that patients who had surveillance contributed person-years to more than a single category of number of surveillance visits.

[b]P values were calculated with the likelihood ratio test.

[c]Multivariable HR adjusted for age, number of premalignant polyps, adenoma histology, adenoma dysplasia, proximal polyps, and length of baseline visit, the characteristics independently associated with CRC incidence in the whole cohort.

[d]High-risk patients were those with ≥2 premalignant polyps of which ≥1 was advanced, ≥5 premalignant polyps, or ≥1 large (≥20mm) non-pedunculated premalignant polyp; low-risk patients had none of these findings.

Supplementary Table 6. Cumulative incidence of colorectal cancer and age-sex-standardised incidence ratios in low-risk patients, without excluding colorectal cancers assumed to have arisen from incompletely excised lesions (n=15,079)

							At 10 years			Standardisation		
	n	(%)	No. of person -years	No. of CRCs	Incidence rate per 100,000 person-years (95% CI)		No. of CRCs	Cumulative incidence (95% CI)[a]	P value[b]	No. of expected CRCs[c]	SIR (95% CI)	
**After baseline (without surveillance, censored at any first surveillance visit)**												
Total	15,079	(100)	90,451	138	153	(129-180)	115	1.6% (1.3-1.9)		182	0.76	(0.64-0.89)
Sex									0.44			
Women	6,796	(45)	42,473	61	144	(112-185)	46	1.3% (1.0-1.8)		68	0.90	(0.69-1.15)
Men	8,283	(55)	47,978	77	160	(128-201)	69	1.8% (1.4-2.4)		114	0.67	(0.53-0.84)
Age at baseline, years									<0.001			
<55	3,469	(23)	22,734	7	31	(15-65)	4	0.2% (0.1-0.6)		11	0.66	(0.26-1.35)
55-64	4,193	(28)	25,273	24	95	(64-142)	20	1.1% (0.7-1.7)		40	0.61	(0.39-0.90)
65-74	4,589	(30)	26,926	65	241	(189-308)	54	2.4% (1.8-3.1)		75	0.87	(0.67-1.10)
≥75	2,828	(19)	15,518	42	271	(200-366)	37	3.1% (2.1-4.4)		57	0.74	(0.53-1.00)
No. of PMPs									0.16			
1	11,733	(78)	70,870	100	141	(116-172)	81	1.4% (1.1-1.8)		140	0.72	(0.58-0.87)
2	2,184	(14)	13,337	24	180	(121-268)	20	1.8% (1.1-2.9)		29	0.83	(0.53-1.24)
3	827	(5)	4,645	9	194	(101-372)	9	2.9% (1.5-5.5)		10	0.86	(0.39-1.64)
4	335	(2)	1,600	5	313	(130-751)	5	4.7% (1.7-12.9)		3	1.46	(0.47-3.40)
PMP size, mm[d]									0.16			
<10	10,985	(73)	69,586	105	151	(125-183)	88	1.6% (1.3-2.0)		140	0.75	(0.61-0.91)
10-19	2,981	(20)	15,651	26	166	(113-244)	20	1.7% (1.0-2.8)		32	0.80	(0.53-1.18)
≥20	1,086	(7)	5,102	6	118	(53-262)	6	1.4% (0.6-3.5)		10	0.60	(0.22-1.31)
Adenoma histology[e]									0.15			
Tubular	10,376	(69)	64,774	88	136	(110-167)	76	1.4% (1.1-1.8)		129	0.68	(0.55-0.84)
Tubulovillous	3,517	(23)	18,944	36	190	(137-263)	28	2.0% (1.3-3.1)		40	0.90	(0.63-1.25)
Villous	359	(2)	1,853	3	162	(52-502)	2	1.1% (0.2-4.5)		4	0.72	(0.15-2.10)
Unknown	827	(5)	4,880	11	225	(125-407)	9	2.7% (1.4-5.5)		9	1.23	(0.61-2.20)
Adenoma dysplasia[f]									0.82			
Low-grade	13,888	(92)	84,243	127	151	(127-179)	105	1.6% (1.3-1.9)		169	0.75	(0.63-0.89)
High-grade	740	(5)	3,321	6	181	(81-402)	6	2.2% (0.9-5.5)		7	0.81	(0.30-1.77)
Unknown	451	(3)	2,887	5	173	(72-416)	4	1.7% (0.6-5.2)		6	0.86	(0.28-2.00)
Proximal polyps[g]									<0.001			
No	9,091	(60)	55,867	65	116	(91-148)	56	1.4% (1.0-1.8)		108	0.60	(0.47-0.77)
Yes	5,988	(40)	34,585	73	211	(168-266)	59	1.9% (1.5-2.5)		75	0.98	(0.77-1.23)

**After first surveillance (with one or more surveillance visits, censored at end of follow-up)**												
Total	7,641	(100)	62,045	70	113	(89-143)	55	1.1% (0.9-1.5)		131	0.54	(0.42-0.68)
Sex									0.09			
Women	3,437	(45)	28,298	39	138	(101-189)	32	1.6% (1.1-2.3)		46	0.85	(0.60-1.16)
Men	4,204	(55)	33,747	31	92	(65-131)	23	0.8% (0.5-1.2)		85	0.37	(0.25-0.52)
Age at baseline, years									0.007			
<55	2,086	(27)	18,864	13	69	(40-119)	10	0.6% (0.3-1.2)		14	0.92	(0.49-1.57)
55-64	2,500	(33)	21,251	22	104	(68-157)	15	0.9% (0.5-1.6)		44	0.50	(0.31-0.76)
65-74	2,251	(29)	17,221	25	145	(98-215)	21	1.7% (1.1-2.7)		55	0.46	(0.29-0.67)
≥75	804	(11)	4,710	10	212	(114-395)	9	2.4% (1.1-5.1)		18	0.56	(0.27-1.03)
No. of PMPs									0.89			
1	5,893	(77)	48,683	53	109	(83-143)	44	1.2% (0.9-1.6)		101	0.53	(0.39-0.69)
2	1,096	(14)	8,396	11	131	(73-237)	7	1.0% (0.5-2.3)		18	0.60	(0.30-1.07)
3	458	(6)	3,464	4	115	(43-308)	3	1.0% (0.3-3.3)		8	0.50	(0.14-1.29)
4	194	(3)	1,502	2	133	(33-532)	1	0.7% (0.1-5.0)		4	0.54	(0.07-1.96)
PMP size, mm[d]									0.43			
<10	5,233	(68)	41,134	45	109	(82-147)	35	1.1% (0.8-1.6)		86	0.52	(0.38-0.70)
10-19	1,674	(22)	14,519	14	96	(57-163)	11	1.0% (0.5-1.8)		32	0.44	(0.24-0.74)
≥20	717	(9)	6,230	11	177	(98-319)	9	1.9% (1.0-3.7)		13	0.85	(0.42-1.51)
Adenoma histology[e]									0.02			
Tubular	5,016	(66)	39,874	33	83	(59-116)	24	0.7% (0.5-1.1)		83	0.40	(0.27-0.56)
Tubulovillous	1,956	(26)	16,197	26	161	(109-236)	22	1.8% (1.1-2.7)		35	0.75	(0.49-1.09)
Villous	217	(3)	1,860	2	108	(27-430)	1	0.7% (0.1-5.0)		4	0.45	(0.05-1.63)
Unknown	452	(6)	4,115	9	219	(114-420)	8	2.7% (1.3-5.4)		9	1.03	(0.47-1.95)
Adenoma dysplasia[f]									0.71			
Low-grade	6,912	(90)	55,214	63	114	(89-146)	49	1.1% (0.8-1.5)		116	0.55	(0.42-0.70)
High-grade	462	(6)	4,059	3	74	(24-229)	3	0.9% (0.3-2.9)		9	0.32	(0.07-0.92)
Unknown	267	(3)	2,772	4	144	(54-384)	3	1.6% (0.5-4.9)		6	0.70	(0.19-1.78)
Proximal polyps[g]									0.23			
No	4,649	(61)	38,524	39	101	(74-139)	31	1.1% (0.7-1.5)		78	0.50	(0.36-0.68)
Yes	2,992	(39)	23,521	31	132	(93-187)	24	1.2% (0.8-1.9)		53	0.59	(0.40-0.83)

CRC=colorectal cancer. CI=confidence interval. SIR=standardised incidence ratio. PMP=premalignant polyp.

Low-risk patients were those without any of the following: ≥2 PMPs of which ≥1 was advanced, ≥5 PMPs, or ≥1 large (≥20mm) non-pedunculated PMP.

[a]Cumulative CRC incidence was estimated using the Kaplan-Meier method.

[b]P values were calculated with the log-rank test to compare cumulative CRC incidence among each category of the specified variable.

[c]Numbers of expected CRCs were calculated by multiplying the 5-year age-group and sex-specific observed person-years by the corresponding CRC incidence rates in the general population of England in 2007.

[d]PMP size was defined according to the largest PMP seen at baseline. Patients with PMPs of unknown size are not included in the table; in the analyses without surveillance, there were 27 such patients, of whom one was diagnosed with CRC; and in the analyses with one or more surveillance visits, there were 17 such patients with no CRC cases.

[e]Adenoma histology was defined according to the greatest degree of villousness seen at baseline.
[f]Adenoma dysplasia was defined according to the highest grade of dysplasia seen at baseline.
[g]Proximal polyps were defined as those proximal to the descending colon.

Supplementary Table 7. Cumulative incidence of colorectal cancer and age-sex-standardised incidence ratios in high-risk patients, without excluding colorectal cancers assumed to have arisen from incompletely excised lesions (n=6,239)

							At 10 years			Standardisation		
	n	(%)	No. of person -years	No. of CRCs	Incidence rate per 100,000 person-years (95% CI)		No. of CRCs	Cumulative incidence (95% CI)[a]	P value[b]	No. of expected CRCs[c]	SIR (95% CI)	
**After baseline (without surveillance, censored at any first surveillance visit)**												
Total	6,239	(100)	25,796	95	368	(301-450)	87	3.7% (2.9-4.8)		60	1.58	(1.28-1.94)
Sex									0.69			
Women	2,226	(36)	9,958	39	392	(286-536)	35	3.9% (2.7-5.7)		18	2.11	(1.50-2.89)
Men	4,013	(64)	15,839	56	354	(272-459)	52	3.6% (2.6-5.0)		42	1.35	(1.02-1.75)
Age at baseline, years									<0.001			
<55	829	(13)	3,983	5	126	(52-302)	5	1.4% (0.6-3.6)		2	2.27	(0.74-5.29)
55-64	1,763	(28)	7,085	17	240	(149-386)	15	3.1% (1.7-5.6)		11	1.53	(0.89-2.45)
65-74	2,305	(37)	8,905	33	371	(263-521)	31	3.7% (2.5-5.5)		25	1.32	(0.91-1.85)
≥75	1,342	(22)	5,823	40	687	(504-936)	36	6.1% (4.2-8.8)		22	1.85	(1.32-2.52)
No. of PMPs									0.53			
1	498	(8)	1,990	8	402	(201-804)	7	3.0% (1.3-6.8)		5	1.66	(0.72-3.28)
2	2,530	(41)	11,638	38	327	(238-449)	34	3.3% (2.2-4.8)		27	1.41	(1.00-1.93)
3	1,208	(19)	4,967	16	322	(197-526)	14	3.4% (1.9-6.2)		12	1.37	(0.79-2.23)
4	616	(10)	2,371	12	506	(287-891)	12	7.0% (3.7-13.1)		6	2.13	(1.10-3.72)
≥5	1,387	(22)	4,830	21	435	(283-667)	20	4.0% (2.4-6.9)		11	1.92	(1.19-2.94)
PMP size, mm[d]									0.002			
<10	568	(9)	2,475	7	283	(135-593)	7	3.2% (1.4-7.3)		5	1.32	(0.53-2.72)
10-19	3,100	(50)	13,757	36	262	(189-363)	32	2.9% (2.0-4.3)		31	1.15	(0.81-1.59)
≥20	2,539	(41)	9,451	52	550	(419-722)	48	5.1% (3.7-7.2)		23	2.25	(1.68-2.95)
Adenoma histology[e]									0.04			
Tubular	2,410	(39)	10,709	31	289	(204-412)	26	3.2% (2.1-4.8)		24	1.30	(0.88-1.84)
Tubulovillous	2,963	(47)	11,753	43	366	(271-493)	41	3.5% (2.4-5.0)		28	1.53	(1.11-2.07)
Villous	686	(11)	2,652	17	641	(398-1,031)	17	6.5% (3.7-11.3)		7	2.54	(1.48-4.06)
Unknown	180	(3)	682	4	587	(220-1,563)	3	7.0% (1.6-27.9)		1	2.96	(0.81-7.57)
Adenoma dysplasia[f]									<0.001			
Low-grade	4,704	(75)	20,157	55	273	(209-355)	49	2.5% (1.8-3.5)		46	1.19	(0.90-1.55)
High-grade	1,408	(23)	5,052	39	772	(564-1,057)	37	8.6% (6.0-12.4)		13	3.06	(2.18-4.18)
Unknown	127	(2)	587	1	170	(24-1,208)	1	5.6% (0.8-33.4)		1	0.93	(0.02-5.19)
Proximal polyps									0.009			
No	2,475	(40)	11,207	29	259	(180-372)	27	2.6% (1.7-3.9)		25	1.15	(0.77-1.66)

Yes	3,764	(60)	14,590	66	452	(355-576)	60	4.7% (3.5-6.3)		35	1.90	(1.47-2.41)
**After first surveillance (with one surveillance visit, censored at any second surveillance visit)**												
Total	3,963	(100)	17,531	57	325	(251-422)	51	4.2% (2.9-6.0)		43	1.34	(1.01-1.73)
Sex									0.48			
Women	1,367	(34)	6,377	19	298	(190-467)	18	4.8% (2.7-8.6)		11	1.67	(1.00-2.61)
Men	2,596	(66)	11,154	38	341	(248-468)	33	3.7% (2.4-5.8)		31	1.21	(0.86-1.67)
Age at baseline, years									0.02			
<55	616	(16)	2,846	9	316	(165-608)	8	2.8% (1.3-6.2)		2	4.79	(2.19-9.09)
55-64	1,299	(33)	5,609	9	160	(83-308)	9	3.0% (1.2-7.7)		11	0.85	(0.39-1.61)
65-74	1,529	(39)	6,684	25	374	(253-554)	21	3.7% (2.2-6.2)		21	1.20	(0.77-1.76)
≥75	519	(13)	2,392	14	585	(347-988)	13	8.6% (4.6-15.9)		9	1.51	(0.82-2.53)
No. of PMPs									0.98			
1	295	(7)	1,308	4	306	(115-815)	4	5.0% (1.6-15.0)		3	1.20	(0.33-3.07)
2	1,521	(38)	7,130	25	351	(237-519)	22	4.6% (2.7-7.8)		17	1.47	(0.95-2.16)
3	767	(19)	3,314	9	272	(141-522)	9	3.4% (1.5-7.7)		8	1.12	(0.51-2.13)
4	402	(10)	1,806	6	332	(149-739)	4	1.6% (0.5-4.8)		5	1.33	(0.49-2.89)
≥5	978	(25)	3,973	13	327	(190-564)	12	5.5% (2.5-11.9)		10	1.33	(0.71-2.28)
PMP size, mm[d]									0.62			
<10	375	(9)	1,637	6	367	(165-816)	6	5.6% (2.0-15.2)		4	1.54	(0.57-3.36)
10-19	1,917	(48)	8,757	24	274	(184-409)	22	3.4% (2.0-5.8)		21	1.15	(0.74-1.71)
≥20	1,649	(42)	7,068	27	382	(262-557)	23	4.8% (2.8-8.2)		18	1.52	(1.00-2.22)
Adenoma histology[e]									0.09			
Tubular	1,510	(38)	6,820	13	191	(111-328)	12	2.2% (1.0-4.4)		16	0.81	(0.43-1.39)
Tubulovillous	1,893	(48)	8,293	33	398	(283-560)	30	6.2% (3.9-9.8)		20	1.62	(1.11-2.27)
Villous	443	(11)	1,896	9	475	(247-912)	8	3.9% (1.9-8.0)		5	1.75	(0.80-3.33)
Unknown	117	(3)	522	2	383	(96-1,533)	1	1.1% (0.2-7.8)		1	1.77	(0.21-6.38)
Adenoma dysplasia[f]									0.05			
Low-grade	2,945	(74)	13,079	34	260	(186-364)	30	3.8% (2.4-6.0)		31	1.09	(0.76-1.52)
High-grade	927	(23)	3,971	20	504	(325-781)	19	5.9% (3.2-10.7)		10	1.92	(1.17-2.96)
Unknown	91	(2)	481	3	623	(201-1,933)	2	3.8% (0.9-15.3)		1	2.75	(0.57-8.04)
Proximal polyps									0.19			
No	1,546	(39)	7,157	19	265	(169-416)	18	3.3% (1.8-5.9)		17	1.14	(0.68-1.77)
Yes	2,417	(61)	10,374	38	366	(267-503)	33	4.8% (3.1-7.5)		26	1.46	(1.04-2.01)
**After second surveillance (with two or more surveillance visits, censored at end of follow-up)**												
Total	2,259	(100)	14,990	33	220	(157-310)	26	2.3% (1.5-3.5)		39	0.85	(0.58-1.19)
Sex									0.50			
Women	741	(33)	5,067	10	197	(106-367)	8	2.1% (1.0-4.3)		9	1.07	(0.51-1.97)
Men	1,518	(67)	9,923	23	232	(154-349)	18	2.5% (1.5-4.2)		30	0.78	(0.49-1.16)
Age at baseline, years									0.04			
<55	402	(18)	3,036	3	99	(32-306)	3	1.6% (0.5-5.1)		3	0.96	(0.20-2.79)

BMJ Publishing Group Limited (BMJ) disclaims all liability and responsibility arising from any reliance placed on this supplemental material which has been supplied by the author(s)

55-64	834	(37)	5,719	10	175	(94-325)	6	1.2% (0.5-3.0)		14	0.72	(0.35-1.32)
65-74	871	(39)	5,450	17	312	(194-502)	14	3.6% (2.0-6.4)		19	0.91	(0.53-1.45)
≥75	152	(7)	785	3	382	(123-1,185)	3	4.9% (1.2-18.4)		3	0.93	(0.19-2.73)
No. of PMPs									0.30			
1	171	(8)	1,266	5	395	(164-949)	4	3.2% (1.2-8.8)		3	1.51	(0.49-3.53)
2	793	(35)	5,100	8	157	(78-314)	5	1.3% (0.5-3.4)		13	0.63	(0.27-1.24)
3	464	(21)	3,092	8	259	(129-517)	6	2.1% (0.9-5.0)		8	0.99	(0.43-1.95)
4	242	(11)	1,576	1	63	(9-450)	1	0.8% (0.1-5.2)		4	0.24	(0.01-1.35)
≥5	589	(26)	3,957	11	278	(154-502)	10	4.1% (2.1-8.1)		11	1.02	(0.51-1.83)
PMP size, mm[d]									0.38			
<10	210	(9)	1,450	3	207	(67-641)	3	2.6% (0.8-8.7)		4	0.81	(0.17-2.37)
10-19	1,063	(47)	6,698	10	149	(80-277)	8	1.2% (0.6-2.4)		17	0.58	(0.28-1.07)
≥20	968	(43)	6,692	20	299	(193-463)	15	3.4% (1.9-5.8)		18	1.14	(0.70-1.76)
Adenoma histology[e]									0.12			
Tubular	854	(38)	5,704	8	140	(70-280)	7	1.2% (0.5-2.6)		15	0.55	(0.24-1.08)
Tubulovillous	1,075	(48)	6,999	15	214	(129-355)	11	2.0% (1.0-3.8)		18	0.82	(0.46-1.35)
Villous	259	(11)	1,697	7	412	(197-865)	7	6.9% (3.1-15.1)		5	1.55	(0.62-3.19)
Unknown	71	(3)	589	3	509	(164-1,578)	1	2.9% (0.4-19.1)		2	1.99	(0.41-5.83)
Adenoma dysplasia[f]									0.79			
Low-grade	1,681	(74)	11,004	23	209	(139-315)	19	2.0% (1.3-3.3)		28	0.82	(0.52-1.22)
High-grade	525	(23)	3,509	8	228	(114-456)	7	3.5% (1.6-7.8)		9	0.85	(0.37-1.67)
Unknown	53	(2)	477	2	419	(105-1,675)	0	-		1	1.50	(0.18-5.41)
Proximal polyps									0.17			
No	853	(38)	5,758	9	156	(81-300)	6	1.2% (0.5-2.8)		15	0.62	(0.28-1.18)
Yes	1,406	(62)	9,232	24	260	(174-388)	20	3.0% (1.8-4.9)		24	0.98	(0.63-1.46)

CRC=colorectal cancer. CI=confidence interval. SIR=standardised incidence ratio. PMP=premalignant polyp.

High-risk patients were those with ≥2 PMPs of which ≥1 was advanced, ≥5 PMPs, or ≥1 large (≥20mm) non-pedunculated PMP.

[a]Cumulative CRC incidence was estimated using the Kaplan-Meier method.

[b]P values were calculated with the log-rank test to compare cumulative CRC incidence among each category of the specified variable.

[c]Numbers of expected CRCs were calculated by multiplying the 5-year age-group and sex-specific observed person-years by the corresponding CRC incidence rates in the general population of England in 2007.

[d]PMP size was defined according to the largest PMP seen at baseline. Patients with PMPs of unknown size are not included in the table; in the analyses without surveillance, there were 32 such patients with no CRC cases; in the analyses with one surveillance visit, there were 22 such patients with no CRC cases; and in the analyses with two or more surveillance visits, there were 18 such patients with no CRC cases.

[e]Adenoma histology was defined according to the greatest degree of villousness seen at baseline.

[f]Adenoma dysplasia was defined according to the highest grade of dysplasia seen at baseline.
